# GITR subverts Foxp3^+^ Tregs to boost Th9 immunity through regulation of histone acetylation

**DOI:** 10.1038/ncomms9266

**Published:** 2015-09-14

**Authors:** Xiang Xiao, Xiaomin Shi, Yihui Fan, Xiaolong Zhang, Minhao Wu, Peixiang Lan, Laurie Minze, Yang-Xin Fu, Rafik M. Ghobrial, Wentao Liu, Xian Chang Li

**Affiliations:** 1 Center for Immunobiology & Transplant Science, Houston Methodist Research Institute, Texas Medical Center, 6670 Bertner Avenue, Houston, Texas 77030, USA; 2 Department of Pathology, University of Chicago, Chicago, Illinois 60637, USA; 3 Department of Surgery, Weill Cornell Medical College of Cornell University, New York, New York 10065, USA

## Abstract

Glucocorticoid-induced TNFR-related protein (GITR) is a costimulatory molecule with diverse effects on effector T cells and regulatory T cells (Tregs), but the underlying mechanism remains poorly defined. Here we demonstrate that GITR ligation subverts the induction of Foxp3^+^ Tregs and directs the activated CD4^+^ T cells to Th9 cells. Such GITR-mediated iTreg to Th9 induction enhances anti-tumour immunity *in vivo*. Mechanistically, GITR upregulates the NF-κB family member p50, which recruits histone deacetylases to the *Foxp3* locus to produce a ‘closed’ chromatin structure. Furthermore, GITR ligation also activates STAT6, and STAT6 renders *Il9* locus accessible via recruitment of histone acetyltransferase p300, and together with inhibition of Foxp3, GITR induces strong Th9 responses. Thus, Th9 cells and iTregs are developmentally linked and GITR can subvert tolerogenic conditions to boost Th9 immunity.

## Abstract (web summary)

Glucocorticoid-induced TNFR-related protein (GITR), a costimulatory protein expressed by T cells, has immunostimulatory effect but the underlying mechanism is not clear. Here the authors show that GITR ligation inhibits the induction of Foxp3 expression and diverts CD4 T cells towards Th9 differentiation instead of iTreg.

## Graphical abstract

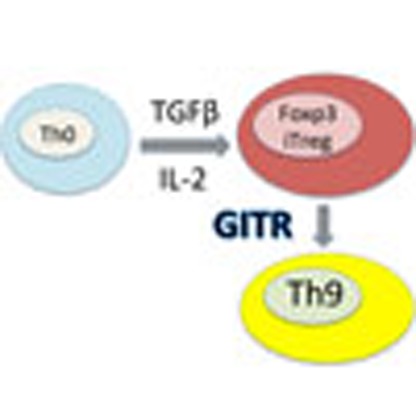

Induction of Foxp3^+^ Tregs from naive CD4^+^ T cells in the periphery (induced regulatory T cells (iTregs)) is a key tolerogenic approach^1^, but the exact mechanisms and the cellular fate of iTregs *in vivo* are poorly understood. In certain models, iTregs can be induced and contribute to immune tolerance^2^,^3^, whereas in others, iTregs lack regulatory functions and may even contribute to tissue pathology^4^,^5^. Moreover, the tumour microenvironment is believed to favour iTregs, which in turn, facilitate tumour evasion^6^. Thus, understanding the fate-decision of iTregs is a therapeutically important issue.

Naive CD4^+^ T cells can also differentiate into functionally distinct T helper subsets upon activation (for example, Th1, Th2, Th17, Th9), as shown by differences in the cytokines they produce^7^. This process is transcriptionally regulated and involves various cytokines and costimulatory signals^8^. Th9 cells are a newly described T-helper subset^9^,^10^; they play an important role in protective immunity^11^, as well as in allergic inflammation^12^,^13^, autoimmune diseases^14^,^15^,^16^ and importantly, in anti-tumour immunity^17^,^18^. Thus, understanding how Th9 cells are induced and regulated is a clinically relevant issue. In contrast to other T helper subsets, Th9 induction requires a constellation of transcription factors, which include SMAD2/3, PU.1, IRF4, STAT5, STAT6, NFAT, GATA1, GATA3, Notch, as well as BATF, RelB/p52 (refs 19, 20), and a single ‘master’ transcription factor has yet been identified in Th9 induction. Furthermore, multiple other cell types including Th2, Th17, natural Tregs and even innate immune cells have been shown to be capable of expressing interleukin (IL)-9 in various models^21^,^22^,^23^,^24^, suggesting the complexity of Th9 induction and potential plasticity of Th9 cells.

Activated CD4^+^ T cells express multiple TNFR superfamily costimulatory molecules, including glucocorticoid-induced TNFR-related protein (GITR), but its contributions to the intricate programs of T-helper cell differentiation process are less well studied. On one hand, naive CD4^+^ T cells do not express GITR under resting state, but GITR is rapidly induced following T-cell receptor stimulation. On the other hand, Foxp3^+^ Tregs constitutively expressed GITR on the cell surface^25^. Studies using GITR-deficient mice or an agonist anti-GITR antibody have shown an immune stimulatory role for GITR in the context of viral infections, tumour immunity and autoimmune diseases^26^,^27^. But it has been difficult to determine the key cell types through which GITR mediates its effects. Controversy over the relative role of GITR on effector versus regulatory T cells in boosting T-cell immunity persists^28^.

In the present study, we examined the mechanisms of GITR costimulation in regulating fate-decisions of CD4^+^ T-helper cells, and found that under iTreg-polarizing conditions, GITR ligation inhibits iTregs and selectively diverts the cells to a Th9 phenotype, which enhances anti-tumour immunity *in vivo*. Mechanistically, we found that GITR signalling controls chromatin remodelling at the *Foxp3* and *Il9* loci via regulation of histone acetylation and deacetylation status, and consequently the suppression of iTregs and induction of Th9 cells.

## Results

### GITR promotes Th9 cells under iTreg-inducing conditions

**Figure 1:**
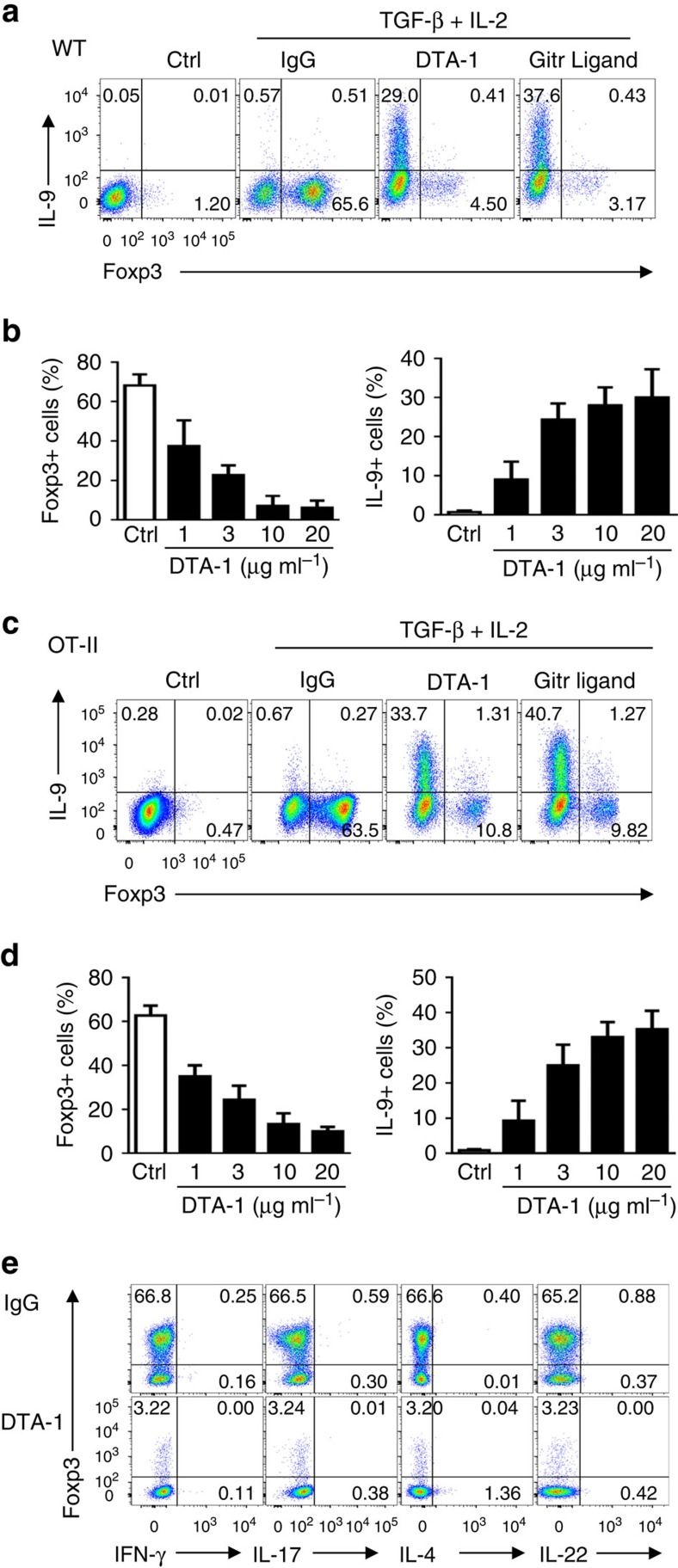
GITR ligation induces Th9 cells under iTreg-polarizing conditions. Naive CD4^+^ T cells were FACS sorted from WT B6 (**a**,**b**) and OT-II (**c**,**d**) *Foxp3gfp* reporter mice and activated under iTreg-polarizing conditions. Anti-GITR agonistic antibody (DTA-1, 10 μg ml^−1^) or His-GITR Ligand (200 ng ml^−1^, along with anti-His mAb) were used to ligate the GITR receptor on activated CD4^+^ T cells. Three days later, cells were harvested and analysed for Foxp3 and cytokines expression by flow cytometry. (**a**,**c**) Colour FACS plots depicting Foxp3- and IL-9-producing cells. Numbers in the quadrants indicate the percentage of cells. Data are representative of five independent experiments. (**b**,**d**) DTA-1 titrations and their impact on induction of Foxp3^+^ Tregs and Th9 cells, and graphs depict the percentage of cells that express Foxp3 (left) or IL-9 (right). Data are pooled from five independent experiments with triplicate cultures. (**e**) Expression of other cytokines, as indicated in the *x* axis, by WT naive CD4^+^ T cells polarized under iTreg conditions for 3 days with or without GITR ligation. Data are representative of three independent experiments.

To determine the role of GITR signalling in regulation of fate-decisions of CD4^+^ T-helper cells, we FACS sorted naive CD4^+^Foxp3^−^ T cells from *Foxp3gfp* reporter mice and activated them *in vitro* with anti-CD3/APC in the presence of transforming growth factor (TGF)-β and IL-2 (iTreg-inducing conditions). As shown in Fig. 1a, a substantial fraction of naive CD4^+^ T cells were converted to Foxp3^+^ T cells 3 days after the culture (∼65%). In these cultures, GITR was highly expressed by CD4^+^ T cells as early as 24 h after activation and maintained for up to 5 days (Supplementary Fig. 1). Interestingly, ligation of GITR on the activated CD4^+^ T cells, using either an agonist mAb DTA-1 or a His-tagged GITR ligand fusion protein (followed by cross-linking with anti-His mAb), markedly inhibited the induction of Foxp3^+^ T cells (down to ∼4%). Unexpectedly, GITR ligation resulted in strong induction of Th9 cells under such iTreg polarizing conditions, and ∼30% of the activated CD4^+^ T cells became IL-9+ Th9 cells (Fig. 1a,b). In another set of experiments, we activated the T-cell receptor transgenic OT-II cells with their cognate antigen OVA presented by autologous APCs, and in this setting, addition of TGF-β and IL-2 in the cultures induced >60% of the OT-II cells to become Foxp3^+^ iTregs 3 days later (Fig. 1c). Again, Foxp3 induction in OT-II cells was also inhibited by GITR stimulation (down to ∼10%) using either DTA-1 mAb or His-tagged GITRL. Similarly, ligation of GITR on the activated OT-II cells under iTreg-polarizing conditions rendered what should be Foxp3^+^ Treg to Th9 cells (30–40%) (Fig. 1c,d). In all experiments, we titrated DTA-1 mAb extensively in the cultures and showed that inhibition of iTregs and induction of Th9 cells by the DTA-1 mAb exhibited a dose-dependent response (Fig. 1b,d).

We also analysed the expression of other T-helper cytokines by GITR stimulated CD4^+^ T cells under the same iTreg-polarizing conditions with flow cytometry (Fig. 1e). GITR ligation did not lead to the induction of interferon (IFN)-γ, IL-4, IL-17 or IL-22, signature cytokines of Th1, Th2, Th17, Th22 subsets, respectively, suggesting that induction of Th9 cells is unique to GITR ligation. Altogether, these data suggest that GITR is uniquely linked to the reciprocal induction of iTregs and Th9 cells, cell types with strikingly different functions *in vivo*.

### GITR triggers chromatin remodelling at *Foxp3* and *Il9* loci

**Figure 2:**
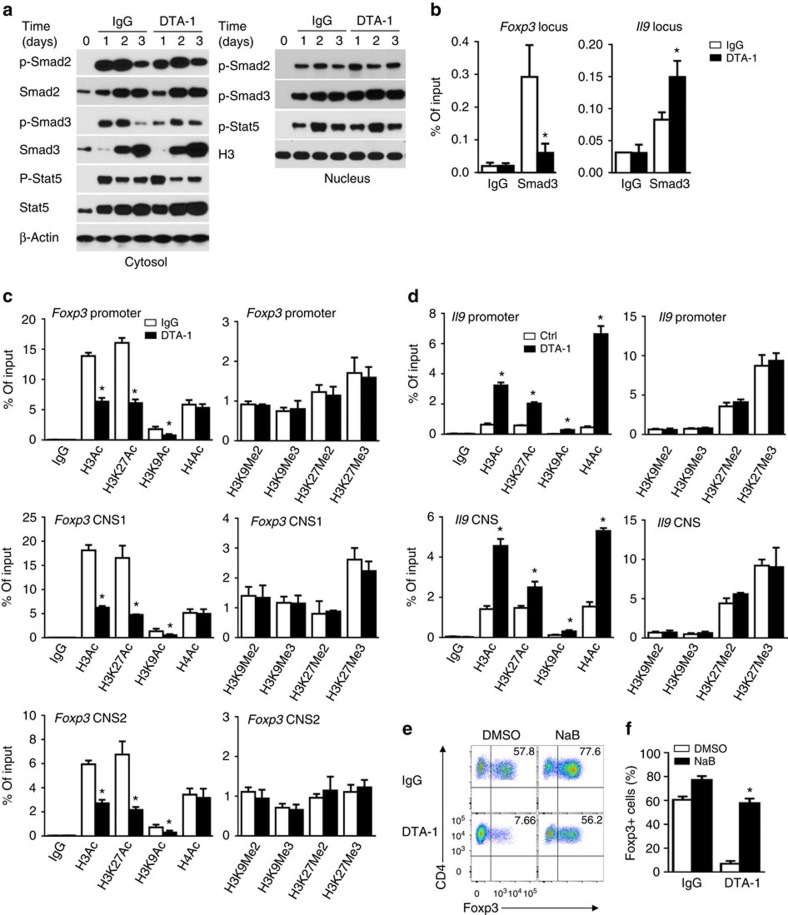
GITR ligation induces extensive chromatin modifications at *Foxp3* and *Il9* loci. (**a**) Immunoblot analysis of total and phosphorylated (p-) Smad2, Smad3 and Stat5 in the cytosol and nucleus in WT B6 naive CD4^+^ T cells left untreated (d0) or activated for 1 to 3 days under iTreg-polarizing conditions with DTA-1 or control IgG. β-actin and Histone 3 (H3) serve as loading controls, respectively. Plots shown are representative data of 3 experiments. (**b**) ChIP assays for Smad3 in *Foxp3* CNS1 and *Il9* promoter regions in WT naive CD4^+^ T cells activated as in **a**. IgG serves as immunoprecipitation control. Values are presented as relative binding based on normalization to input DNA. Data represent mean values±s.d. (*n*=3). (**c**,**d**) ChIP assays for H3Ac, H3K27Ac, H3K9Ac, H4Ac, H3K9Me2, H3K9Me3, H3K27Me2 and H3K27Me3 modifications in *Foxp3* (**c**) and *Il9* (**d**) loci in WT naive CD4^+^ T cells activated as in (**b**) for 2 days. Data represent mean values±s.d. (*n*=3). (**e**,**f**) Flow cytometry analysis of Foxp3 expression in WT naive CD4^+^ T cells activated as in (**b**) for 3 days in the presence or absence of HDAC inhibitor sodium butyrate (NaB, 250 μg ml^−1^). Numbers in the quadrants indicate the percentage of Foxp3^+^ cells (**e**). (**f**) The graph represents Mean±s.d. of Foxp3^+^ T cells from five experiments with triplicate cultures. *P* values were determined by Student’s *t*-test (*P<0.05).

The induction of Th9 cells by GITR under iTreg-inducing conditions is intriguing. Of particular interest is that TGF-β and IL-2 induce Foxp3^+^ Tregs through activation of SMAD2/3 and STAT5, transcription factors that are also implicated in Th9 induction^29^. To gain further insights, we analysed the signalling process of polarizing cytokines, transcription status of *Foxp3* and *Il9* loci, as well as epigenetic changes at the *Foxp3* and *Il9* loci in CD4^+^ T cells polarized under iTreg conditions with or without GITR ligation. As shown in Fig. 2a, expression of SMAD2, SMAD3, STAT5, their phosphorylation status, as well as their nuclear localization were all comparable in activated CD4^+^ T cells regardless of GITR ligation, showing the same kinetics and levels of expression (within the first 3 days examined), suggesting that GITR ligation unlikely alters the cytokine signalling pathways to impart reciprocal induction of iTreg and Th9 cells.

We then used SMAD3 as a reporter molecule to probe the accessibility of *Foxp3* (ref. 30) and *Il9* loci (Supplementary Fig. 2) in GITR stimulated CD4^+^ T cells activated under iTreg conditions. Chromatin immunoprecipitation (ChIP) analysis showed that SMAD3 was clearly enriched at the CNS1 region of *Foxp3* locus, and GITR stimulation markedly reduced the binding of SMAD3 to the *Foxp3* locus (Fig. 2b). Interestingly, GITR stimulation enhanced the binding of SMAD3 to the *Il9* locus (Fig. 2b), as compared with those activated without GITR stimulation. Thus, GITR ligation affects the accessibility of *Foxp3* and *Il9 loci* to key transcription factors. To further examine this issue, we analysed side by side the acetylation and methylation status of *Foxp3* locus, which include the promoter, CNS1, and CNS2 regions^30^, in CD4^+^ T cells polarized under iTreg conditions with or without GITR ligation, and compared with those of *Il9* locus in identically treated CD4^+^ T cells. As shown in Fig. 2c,d, we observed striking differences in histone acetylation status at the *Foxp3* and *Il9* loci. Specifically, in the *Foxp3* promoter, CNS1, and CNS2 regions, histone 3 (H3), as well as H3K27 and H3K9 were hyperacetylated in CD4^+^ T cells activated under iTreg conditions, and this histone hyperacetylation was markedly inhibited by GITR stimulation, leading to hypoacetylation at H3 and H3K27 sites (Fig. 2c). The acetylation status of H4 did not change with or without GITR ligation. The methylation status of H3, including H3K9 and H3K27, did not show any differences in the *Foxp3* locus in GITR stimulated CD4^+^ T cells activated under iTreg conditions (Fig. 2c, right panels).

The changes at the *Il9* locus are mirror opposite of those at the *Foxp3* locus following GITR stimulation (Fig. 2d). GITR stimulation resulted in hyperacetylation of H3 and H3K27 at the promoter region and CNS region of *Il9* locus, as compared with CD4^+^ T cells activated without GITR ligation. Unlike in the *Foxp3* locus, GITR stimulation resulted in hyperacetylation of H4 (Fig. 2d), but similar to that of *Foxp3* locus, GITR ligation did not change the histone methylation status in the *Il9* locus (Fig. 2d, right panels). Taken together, these data suggest that under iTreg-polarizing conditions, GITR ligation controls the histone acetylation and deacetylation status of *Foxp3* and *Il9* loci.

In most cases, histone deacetylation is associated with gene repression^31^. To test whether histone deacetylation at the *Foxp3* locus is functionally involved in suppression of Foxp3 expression by GITR, we activated naïve CD4^+^ T cells under iTreg conditions together with GITR stimulation, and in these cultures, we added a pan-histone deacetylase inhibitor sodium butyrate (NaB) to inhibit deacetylase activities^32^,^33^. As shown in Fig. 2e,f, addition of NaB in the cultures markedly rescued Foxp3 from GITR-mediated suppression, and ∼60% of the activated CD4^+^ T cells became Foxp3^+^ T cells in spite of GITR ligation, and this is associated with increased H3 acetylation at the Foxp3 locus (Supplementary Fig. 3), demonstrating the importance of histone deacetylation in GITR-mediated suppression of Foxp3 induction.

### Critical role of p50 in GITR-mediated suppression of iTregs

**Figure 3:**
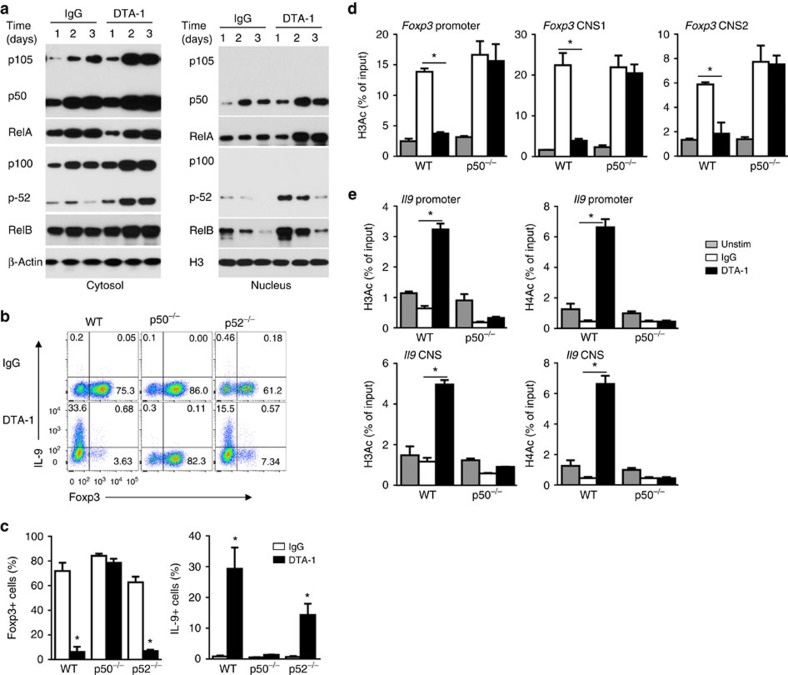
Role of the NF-κB pathways in GITR-mediated suppression of iTregs. (**a**) Immunoblot analysis of the induction of the canonical (p50, RelA) and non-canonical (p52, RelB) NF-κB pathways in the cytosol and nucleus in WT B6 naive T cells activated for 1 to 3 days under iTreg-polarizing conditions with DTA-1 or Ctrl IgG. Data are representative of three independent experiments. (**b**,**c**) Naive CD4^+^ T cells from WT B6, p50^−/−^ and p52^−/−^ mice were activated under iTreg conditions, with or without GITR ligation for 3 days, and induction of Foxp3^+^ T cells and IL-9+ T cells was analysed by FACS and shown. Numbers in the quadrants indicate the percentage of cells. (**c**) Graphs depict Mean±s.d. of five experiments with triplicate cultures. (**d**,**e**) ChIP analysis of H3Ac and H4Ac modifications at *Foxp3* (**d**) and *Il9* (**e**) promoter and CNS regions from naive WT and p50^−/−^ CD4^+^ T cells, either left untreated (Unstim) or activated under iTreg-polarizing conditions for 2 days in the presence of DTA-1 or Ctrl IgG. Data represent mean values±s.d. (*n*=3). *P* values were determined by Student’s *t*-test (*P<0.05).

To determine how GITR ligation inhibits histone acetylation at *Foxp3* locus, we first examined signalling molecules induced by GITR ligation in CD4^+^ T cells under iTreg conditions. As shown in Fig. 3a, activation of CD4^+^ T cells in the presence of GITR agonist mAb DTA-1 activated both the canonical (p50/RelA) and the non-canonical (p52/RelB) NF-κB pathways. Moreover, nuclear localization of such transcription factors was also markedly increased in GITR stimulated CD4^+^ T cells (Fig. 3a, right panel). To examine the relative importance of such two pathways in regulating induction of iTregs and Th9 cells, we sorted naive CD4^+^Foxp3^−^ T cells from p50KO and p52KO mice, in which activation of the canonical and the non-canonical NF-κB pathways is genetically abrogated, respectively^34^,^35^, and compared the induction of iTregs and Th9 cells with that from wild-type (WT) B6 mice with or without GITR ligation. As shown in Fig. 3b, deficiency of p50 completely abolished the effect of GITR in suppression of iTregs, and all p50KO T cells remained Foxp3^+^ (∼80%) regardless of GITR ligation, and surprisingly, GITR also failed to convert p50KO cells to Th9 cells (Fig. 3b), which is in stark contrast to WT CD4^+^ T cells. We observed that p52KO CD4^+^ T cells could be readily converted to Foxp3^+^ T cells (>60%), and similar to WT CD4^+^ T cells, GITR ligation inhibited the induction of Foxp3^+^ T cells from p52KO CD4^+^ T cells. GITR ligation also converted p52KO CD4^+^ cells to IL-9 producing cells, though at a lower level (Fig. 3b,c). In all those experiments, GITR expression by activated WT B6, p50KO and p52KO CD4^+^ T cells was comparable at different time points following cell activation, as revealed by flow cytometry (Supplementary Fig. 4). These data pinpoint the importance of p50 in GITR-mediated control of iTregs and Th9 cells.

In ChIP assays using acetylated H3 and H4 (H3Ac, H4Ac) as ‘permissive’ chromatin marks, we compared the accessibility of *Foxp3* and *Il9* loci in WT B6 and p50KO CD4^+^ T cells activated under iTreg conditions with or without GITR ligation. As shown in Fig. 3d,e (Supplementary Fig. 5), in WT B6 CD4^+^ T cells, GITR enabled ‘permissive’ histone marks in *Il9* locus (hyperacetylated H3 and H4), and conversely, a ‘non-permissive’ histone mark in *Foxp3* locus (hypoacetylated H3), when compared with cells without GITR ligation. In p50KO CD4^+^ T cells, however, GITR ligation failed to induce hyperacetylation of H3 and H4 in *Il9* locus and hypoacetylation of H3 in *Foxp3* locus (closed histone mark), which correlated with the failure of GITR ligation in suppression of Foxp3^+^ cells and induction of Th9 cells from p50KO CD4^+^ T cells (Fig. 3b).

### NF-kB p50 recruits histone deacetylases to the *Foxp3* locus

**Figure 4:**
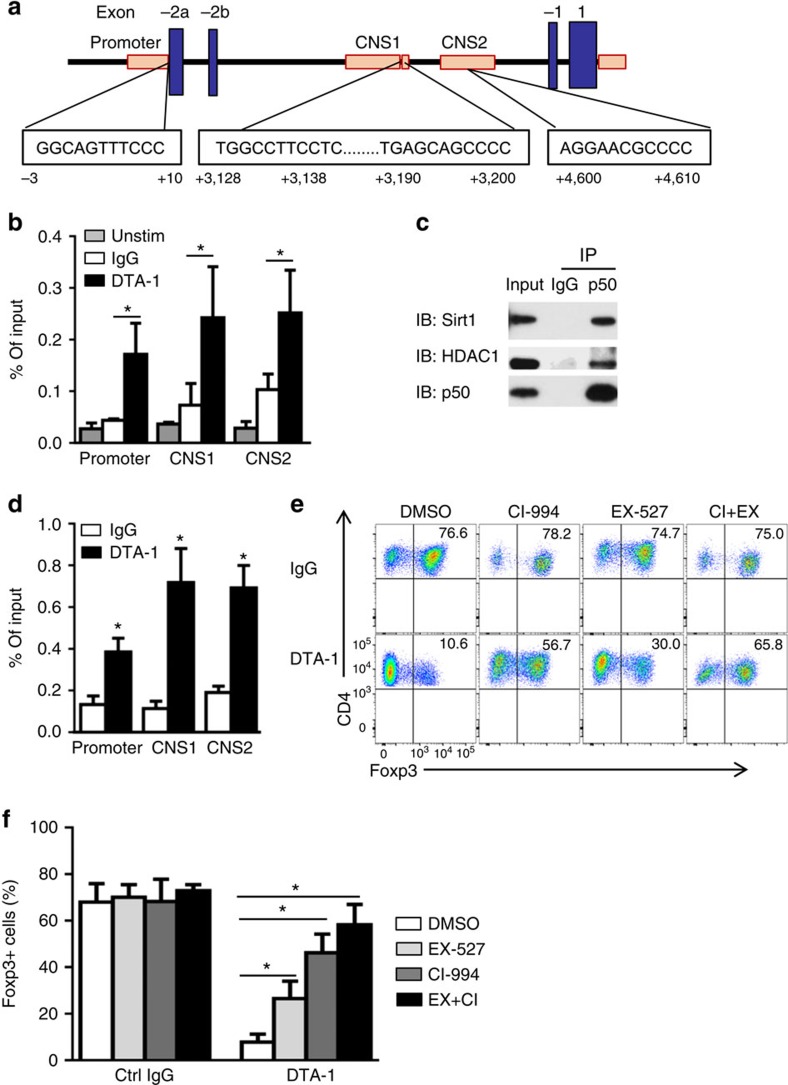
Role of p50 in recruitment of histone deacetylases to the *Foxp3* locus to inhibit Foxp3 expression. (**a**) Schematic diagram depicting *Foxp3* locus structure and consensus κB binding sites (boxed) in the *Foxp3* promoter and CNS1–2 regions. (**b**) ChIP analysis of p50 enrichment at the *Foxp3* promoter and CNS1–2 sites in naive CD4^+^ cells left untreated (Unstim) or activated under iTreg-polarizing conditions in the presence of DTA-1. Data represent mean values±s.d. (*n*=4). (**c**) Co-immunoprecipitation analysis of p50 in naive CD4^+^ T cells activated as in (**b**). Anti-p50 or control IgG immunoprecipitates (IP) were subjected to immunoblot analysis (IB) using anti-HDAC1 and anti-Sirt1 antibodies. Data are representative of three independent experiments. (**d**) ChIP analysis of HDAC1 binding to the *Foxp3* promoter and CNS1–2 sites in naive CD4^+^ cells stimulated under iTreg-polarizing conditions in the presence of DTA-1 or IgG. Data represent mean values±s.d. (*n*=3). (**e**,**f**) Flow cytometry analysis of Foxp3 expression in naive CD4^+^ T cells activated as in (**d**) for 3 days in the presence of HDAC1 inhibitor CI-994 (CI, 1 μM) or Sirt1 inhibitor EX-527 ( EX, 0.5 μM). Numbers in the quadrants indicate the percentage of Foxp3^+^ cells (**e**). (**f**) Plots shows mean±s.d. of Foxp3^+^ T cells from 3 experiments with duplicate culture. *P* values were determined by Student’s *t*-test (*P<0.05).

Analysis of the *Foxp3* locus revealed multiple κB consensus binding sites (Fig. 4a), which is in line with a previous report^36^, and these κB consensus sites may potentially engage NF-κB family transcription factors. Indeed, ChIP assay showed that in WT B6 CD4^+^ T cells activated in the presence of GITR stimulation, p50 was highly enriched at *Foxp3* promoter, CNS1 and CNS2 regions (Fig. 4b), areas that are also extensively deacetylated on GITR ligation (Fig. 2c). As p50 does not possess any deacetylase activities, but is capable of partnering with many other molecules^37^, we reasoned that p50 may bind to the κB sites, and through recruitment of histone deacetylases to the *Foxp3* locus, p50 may control histone acetylation status. To test this possibility, we immunoprecipitated p50 from GITR stimulated CD4^+^ T cells activated under iTreg polarizing conditions, and performed extensive immunoblotting experiments to identify known histone deacetylases. We observed that p50 could physically interact with HDAC1 and Sirt1, as HDAC1 and Sirt1, but not other HDACs, could be readily detected in p50 immunoprecipitates (Fig. 4c). Furthermore, we also showed that HDAC1 was selectively enriched at *Foxp3* locus in GITR stimulated CD4^+^ T cells, as compared with CD4^+^ T cells activated without GITR ligation, as shown by ChIP assays (Fig. 4d). We were unable to assess Sirt1 because of the lack of ChIP grade mAbs. In another set of experiments, addition to HDAC1 and Sirt1 specific inhibitors in the cultures (CI-994 and EX-527)^38^,^39^ markedly rescued Foxp3 from GITR-mediated suppression ( from ∼10 to ∼65% Foxp3^+^ T cells; Fig. 4e,f), thus demonstrating a critical role for histone deacetylases in GITR-mediated suppression of Foxp3. Collectively, these data suggest that GITR ligation induces p50, which recruits HDAC1 and Sirt1 to *Foxp3* locus where they mediate histone deacetylation, and consequently, ‘close’ the *Foxp3* locus.

### Foxp3 is a potent repressor of *Il9* gene expression

**Figure 5:**
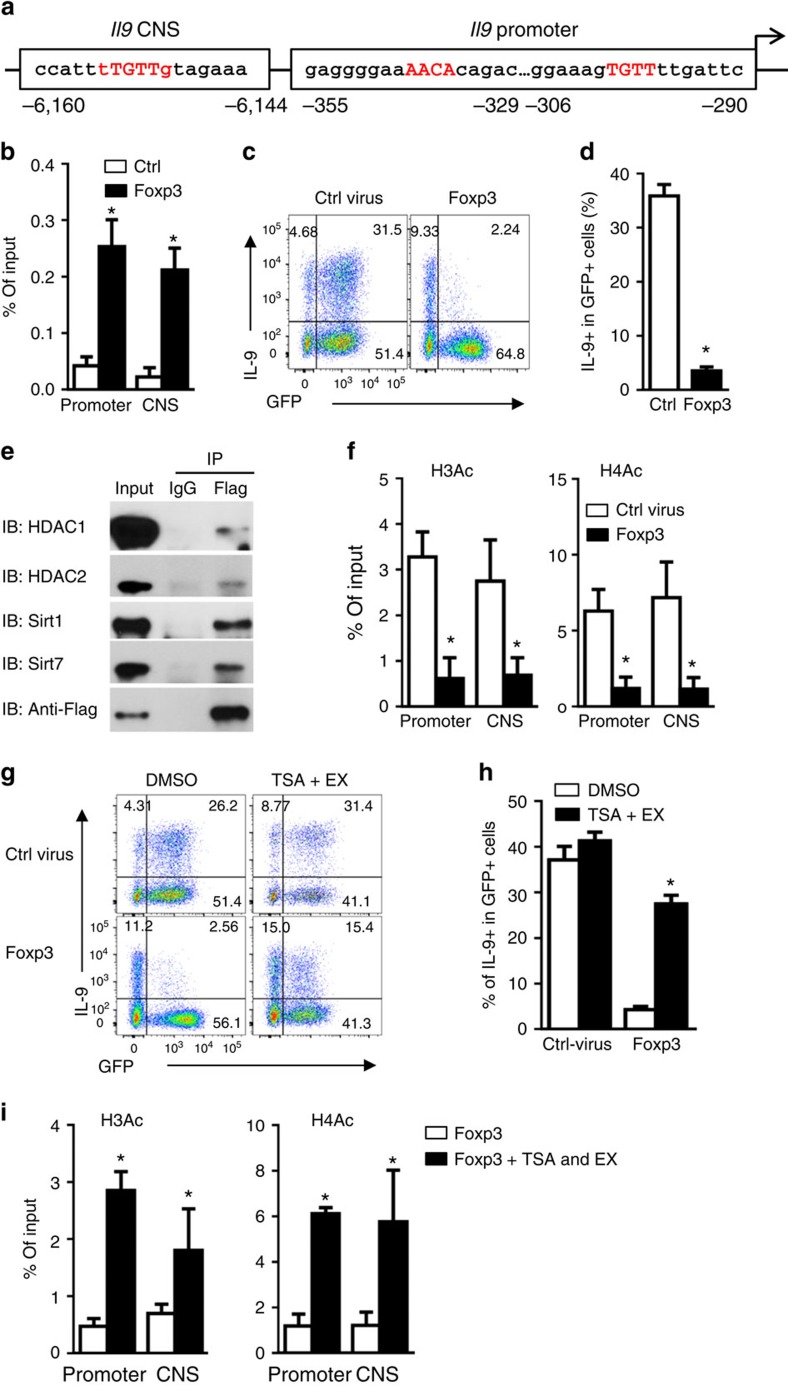
Foxp3 binds to the *Il9* locus and inhibits IL-9 expression by recruiting histone deacetylases. (**a**) Graph depicting *Il9* locus and putative Foxp3 binding sites (red) at the *Il9* promoter and CNS regions. (**b**) ChIP analysis of Foxp3 binding to the *Il9* gene locus in CD4^+^ T cells transduced with retrovirus expressing GFP alone (Ctrl) or GFP-Flag-Foxp3 (Foxp3), then cultured under iTreg-polarizing conditions in the presence of DTA-1. GFP^+^ cells were sorted and fixed for ChIP using anti-Flag antibody. Data represent mean values±s.d. (*n*=3). (**c**,**d**) Flow cytometry plots showing IL-9-producing cells in CD4^+^ T cells transduced with retrovirus expressing GFP alone or Foxp3-GFP, and cultured under iTreg-polarizing conditions for 3 days in the presence of DTA-1. Numbers indicate the percentage of cells in the quadrants (**c**). Graph depicts the percentage of IL-9+ cells in GFP^+^ population (**d**). Data represent mean values±s.d. (*n*=6). (**e**) Co-immunoprecipitation of flag-foxp3 in CD4^+^ T cells transduced with retrovirus expressing flag-foxp3, and cultured under iTreg-polarizing conditions for 2 days. Anti-flag and control IgG immunoprecipitates (IP) were subjected to immunoblot analysis (IB) with anti-HDAC1, anti-HDAC2, anti-Sirt1 and anti-Sirt7 antibodies, and representative plots of 3 experiments are shown. (**f**) ChIP analysis of H3Ac and H4Ac modifications at *Il9* promoter and CNS regions in GFP^+^ cells sorted from naive CD4^+^ T cells transduced and activated as in (**b**) for 2 days. Data represent mean values±s.d. (*n*=3). (**g**,**h**) Flow cytometry analysis of IL-9-producing cells in naive CD4^+^ T cells transduced and activated as in (**c**) for 3 days in the presence or absence of HDAC inhibitor Trichostatin A (TSA, 2 nM) and Sirt1 inhibitor EX-527 (EX, 0.5 μM). Numbers in the quadrants indicate the percentage of positive cells (**g**). Graph depicts the percentage of IL-9+ cells in GFP^+^ population. Data represent mean values±s.d. (*n*=9) (**h**). (**i**) ChIP analysis of H3Ac and H4Ac modifications at *Il9* promoter and CNS regions in Foxp3^−^GFP^+^ cells sorted from naive CD4^+^ T cells transduced with retrovirus expressing Foxp3-GFP and cultured as in (**g**) for 2 days. Data represent mean values±s.d. (*n*=3). *P* values were determined by Student’s *t*-test (*P<0.05).

In our experiments, expression of Foxp3 and IL-9 is mutually exclusive. Analysis of the *Il9* locus revealed binding sites for Foxp3 (Fig. 5a), in addition to binding sites for SMAD2/3, IRF4, PU.1, STAT6, BATF, transcription factors that are key to Th9 induction^9^, which is relevant to differential induction of iTregs and Th9 cells by GITR. Indeed, ChIP assay revealed that Foxp3 was highly enriched at the *Il9* promoter and CNS regions in CD4^+^ T cells polarized under iTreg conditions (Fig. 5b), suggesting a potential role of Foxp3 in repressing Th9 induction. To further examine this possibility, we overexpressed Foxp3 in activated CD4^+^ T cells using the retroviral-mediated gene transfer method, and further stimulated the CD4^+^ T cells under iTreg-polarizing condition in combination with GITR ligation. As shown in Fig. 5c, as compared with control viral-transduced cells (GFP^+^ fraction) in which IL-9 was highly induced (∼32% IL-9+ cells), overexpression of Foxp3 completely inhibited IL-9 expression in the GFP^+^ fraction (∼2%; Fig. 5c,d). Interestingly, in a fraction of T cells that did not express GFP, and therefore Foxp3^−^, IL-9 expression remained inducible (Fig. 5c), demonstrating that Foxp3 is a potent repressor of *Il9* gene expression.

We genetically introduced a *Flag* tag to the *Foxp3* gene, and then transfected the Flag-tagged *Foxp3* construct into activated CD4^+^ T cells. The transduced cells were activated under iTreg-inducing conditions in the presence of GITR ligation. Two days later, we imunoprecipitated Foxp3 from iTreg-polarized CD4^+^ T cells using anti-FLAG mAb, and co-immunoprecipitation analysis identified the presence of HDAC1/2 and Sirt1/7 in the Foxp3 precipitates (Fig. 5e), suggesting that Foxp3 could also recruit histone deacetylases to the *Il9* locus to close *Il9* locus. Indeed, ChIP analysis showed that in CD4^+^ T cells that overexpress Foxp3, H3 and H4 in the promoter and the CNS regions of *Il9* locus were markedly hypoacetylated (Fig. 5f), and addition of HDAC1/2 and Sirt1 inhibitors (TSA and EX-527)^40^ during iTreg polarization inhibited hypoacetylation of H3 and H4 at *Il9* locus, and consequently, rescued IL-9 from Foxp3-mediated suppression (Fig. 5g,h). Furthermore, IL-9 expression in the presence of TSA and EX-527 was correlated with increased H3 and H4 acetylation at the *Il9* locus (Fig. 5i). Altogether, these data suggest that inhibition of *Foxp3* expression seems necessary for successful Th9 induction.

### GITR activates STAT6 to mediate Th9 induction

**Figure 6:**
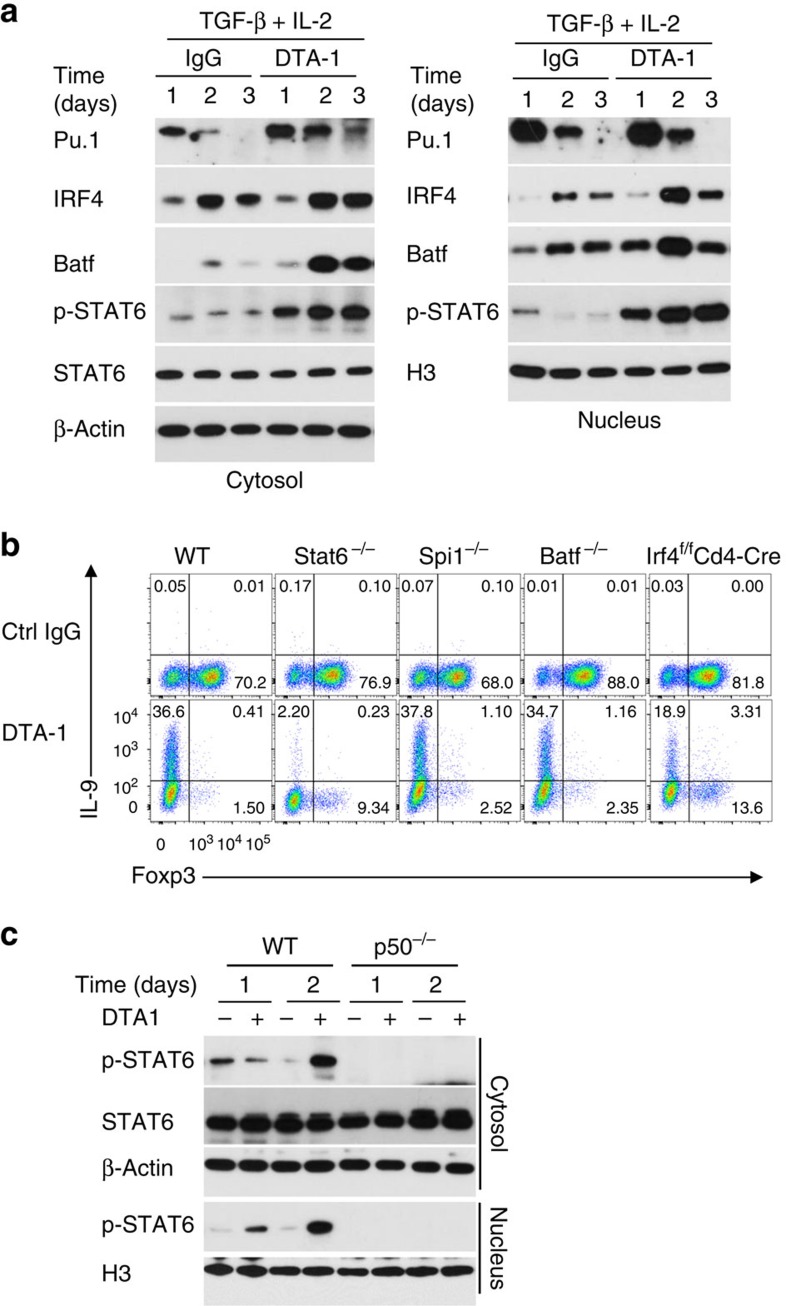
Role of Th9 associated transcription factors in GITR-mediated induction of iTregs and Th9 cells. (**a**) Immunoblot analysis of PU.1, IRF4, BATF, STAT6, and p-STAT6 in WT naive CD4^+^ T cells activated for 1 to 3 days under iTreg-polarizing conditions with DTA-1 or control IgG. Data shown are representative of two independent experiments. (**b**) Induction of Foxp3^+^ T cells and IL-9+ T cells from WT B6, Stat6^−/−^, Spi1^−/−^, Irf4^f/f^Cd4-Cre, and Batf ^−/−^ CD4^+^ T cells activated under iTreg conditions for 3 days with or without GITR ligation. Numbers in the quadrants indicate the percentage of cells. Data are representative of five independent experiments. (**c**) Immunoblot analysis of STAT6 and p-STAT6 in WT B6 and p50^−/−^ naive CD4^+^ T cells activated for 1 to 2 days under iTreg-polarizing conditions with DTA-1 or Ctrl IgG. Data are representative of two independent experiments.

To further examine whether GITR triggers other pathways in directing iTregs to Th9 cells, we performed extensive immunoblotting to assess expression of Th9 associated transcription factors in GITR stimulated CD4^+^ T cells under iTreg culturing conditions^19^. We observed that in GITR stimulated CD4^+^ T cells, expression of PU.1, IRF4 and BATF3 and phosphorylation of STAT6 were noticeably upregulated, either in the cytosol or in the nucleus, as compared to that in CD4^+^ T cells activated without GITR ligation (Fig. 6a), although levels of expression varied among individual factors. Studies using genetic knockout mice showed that naïve CD4^+^ T cells sorted from mice deficient for *Spi1*, *Irf4*, *Batf* or *Stat6* could be converted to Foxp3^+^ Tregs by TGF-β and IL-2, in a manner that is similar to WT B6 CD4^+^ T cells (∼70 to 80% Foxp3^+^ T cells; Fig. 6b). In addition, they expressed comparable levels of GITR on the cell surface on activation, as assessed by flow cytometry (Supplementary Fig. 6). In these cultures, we observed that deficiency of PU.1, BATF, or IRF4 did not alter the ability of GITR in suppression of Foxp3^+^ Tregs as well as in promotion of Th9 cells, and similar to the effects in WT CD4^+^ T cells (Fig. 6b). In stark contrast, GITR ligation failed to convert STAT6KO CD4+ T cells to Th9 cells, despite marked inhibition of Foxp3 expression (Fig. 6b), suggesting that Foxp3 inhibition alone, though necessary, is not sufficient in Th9 induction by GITR stimulation. Interestingly, in p50KO CD4^+^ T cells, GITR ligation completely failed to activate STAT6, as shown by STAT6 phosphorylation (Fig. 6c), indicating that activation of STAT6 by GITR also requires p50.

### STAT6 recruits histone acetyltransferase p300 to open *Il9* locus

**Figure 7:**
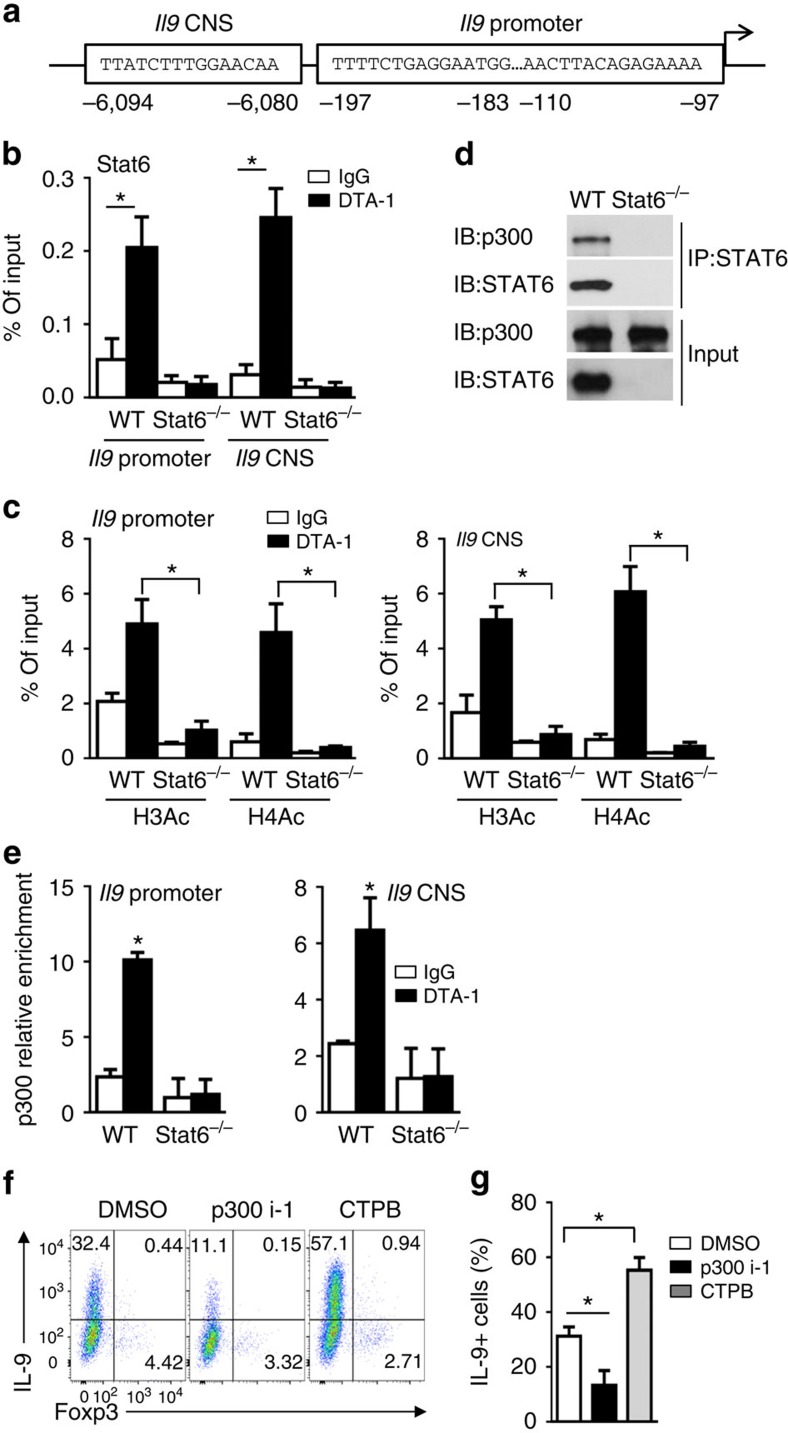
Role of STAT6 and P300 in GITR-mediated hyperacetylation of *Il9* locus. (**a**) Graph showing the *Il9* locus and putative STAT6 binding sites at the *Il9* promoter and CNS regions. (**b**) ChIP analysis of the binding of STAT6 to *Il9* locus in WT B6 and Stat6^−/−^ naive CD4^+^ T cells activated under iTreg-polarizing conditions for 2 days in the presence of DTA-1 or control IgG. Data are representative of three independent experiments. (**c**) ChIP analysis of H3Ac and H4Ac modifications in *Il9* promoter (top) and CNS regions (bottom) in WT B6 and Stat6^−/−^ naive CD4^+^ T cells activated as in (**b**) for 2 days. Data are representative of three independent experiments. (**d**) Co-immunoprecipitation analysis of STAT6 in WT B6 and Stat6^−/−^ naive CD4^+^ T cells activated as in (**b**). Anti-STAT6 immunoprecipitates (IP) were subjected to immunoblot analysis (IB) with anti-P300 and anti-STAT6 antibodies. Data are representative of three independent experiments. (**e**) ChIP analysis of p300 binding to the *Il9* promoter and CNS in WT B6 and Stat6^−/−^ naive CD4^+^ T cells activated as in (**b**) for 2 days. Data are representative of three independent experiments. (**f**,**g**) Flow cytometry analysis of IL-9 and Foxp3 expression by WT B6 naive CD4^+^ T cells activated as in Fig. 5g for 3 days in the presence or absence of p300/CBP inhibitor 1 (p300i-1, 4 μM) or p300 HAT activator (CTPB, 50 μM). Numbers in the quadrants indicate the percentage of cells (**f**). The graph represents Mean±s.d. of IL-9^+^ T cells from three independent experiments with triplicate cultures (**g**). *P*-values were determined by Student’s *t*-test (*P<0.05).

In the *Il9* promoter and CNS regions, there are multiple STAT6 binding sites (Fig. 7a), and STAT6, among others, is critical to Th9 induction^41^. But how STAT6 works remains unknown. ChIP assay showed that GITR ligation on CD4^+^ T cells activated under iTreg condition induced STAT6 enrichment at the promoter and CNS regions of *Il9* locus (Fig. 7b), which is correlated with the impaired Th9 induction in STAT6KO T cells by GITR (Fig. 6b). We again used acetylated H3 and H4, (H3Ac, H4Ac) as ‘permissive’ chromatin marks to assess *Il9* locus accessibility in WT B6 and STAT6KO CD4^+^ T cells activated with or without GITR stimulation. As shown in Fig. 7c, in STAT6KO CD4^+^ T cells GITR failed to induce H3 and H4 hyperacetylation in the *Il9* locus, which contrasts to that in WT CD4^+^ T cells, suggesting that the *Il9* locus fails to be acetylated without STAT6, and therefore, remains inaccessible. In a co-immunoprecipitation experiment, we showed that p300, a histone acetyltransferase, interacted with STAT6 in GITR stimulated WT CD4^+^ T cells (Fig. 7d). Consistently, we also showed that p300 was selectively enriched at *Il9* locus in GITR stimulated CD4^+^ T cells, as compared with CD4^+^ T cells activated without GITR ligation, and STAT6 is required in this process, as GITR failed to induce p300 enrichment at *Il9* locus in Stat6-deficient cells (Fig. 7e). Taken together, our data demonstrate that STAT6 is crucial in recruiting p300 to the *Il9* locus by GITR ligation. To further ascertain this notion, in WT B6 CD4^+^ T cells activated under iTreg conditions in the presence of GITR ligation, we added either a p300 inhibitor (p300i-1) or a p300 activator (CTPB) to selectively modulate the p300 activity, and then determined the impact on iTreg and Th9 induction. As shown in Fig. 7f,g, GITR ligation consistently inhibited the induction of Foxp3^+^ T cells, inhibition of p300 markedly reduced Th9 cells (to ∼10%), whereas activation of p300 strongly enhanced the induction of Th9 cells (∼57%), thus providing strong evidence on the importance of p300 in Th9 induction. Collectively, our data demonstrate that under iTreg-polarizing conditions, GITR activates p50 to shut down Foxp3 through recruitment of HDAC; it also activates STAT6/p300 to render *Il9* locus accessible. Both are required in Th9 induction under iTreg-inducing conditions.

### GITR induces Th9-mediated anti-tumour immunity *in vivo*

**Figure 8:**
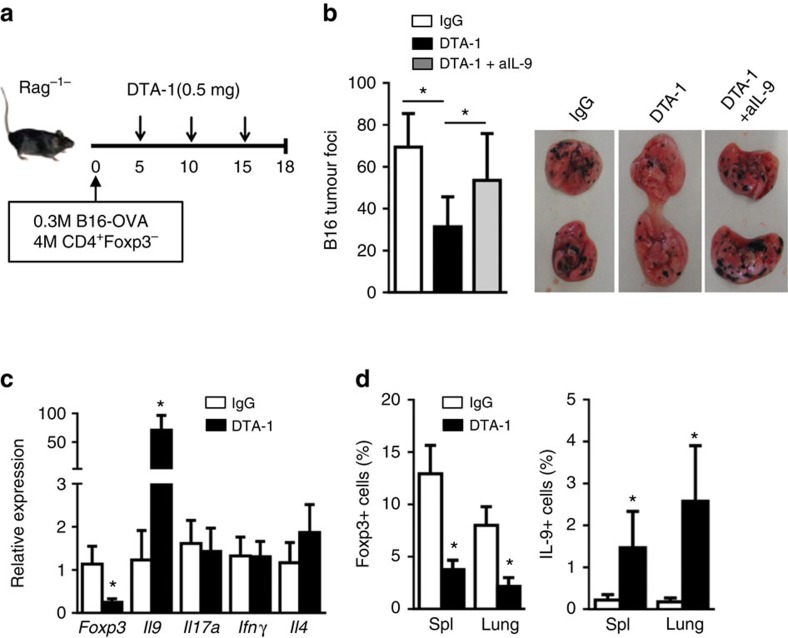
GITR ligation induces Th9-mediated anti-tumour immunity *in vivo.* (**a**) Rag1^−/−^ mice were injected with B16-OVA melanoma cells together with sorted CD4^+^ T cells from OT-II mice. The host mice were treated with 3 doses of DTA-1 (0.5 mg, i.p., *n*=14) or control IgG (*n*=14) every five days starting from Day 5 after B16 inoculation. A cohort of anti-GITR treated Rag-1−/− mice (*n*=7) was also given a neutralizing anti-IL-9 mAb (0.2 mg, i.p.). All animals were sacrificed on day 18 for analysis. (**b**) Numbers of tumor foci in the lungs from mice treated as in (**a**) (left panel). Representative images of the lungs of mice receiving different treatments (right panel). (**c**) Quantitative RT–PCR analysis of *Foxp3, Il9, Il17a, IFNγ* and *Il4* mRNA from the lung infiltrating CD4^+^ cells. Data represent mean values±s.d. (*n*=6). (**d**) Percentage of Foxp3^+^ (left) or IL-9^+^ (right) cells among the lung infiltrating CD4^+^ cells and in host spleen from control IgG and DTA treated mice as assessed by flow cytometry. Data represent mean values±s.d. (*n*=6). *P*-values were determined by Student’s *t*-test (*P<0.05).

We used a melanoma tumour model in which Rag-1^−/−^ mice were adoptively transferred with OT-II T cells (4 million per mouse; Fig. 8a), and observed that seeding the mice i.v. with B16-OVA melanoma cells (0.3 million cells per mouse) resulted in widespread tumour nodules in the lungs 18 days after tumour cell inoculation, with an average of ∼70 foci (Fig. 8b). Treatment of the host mice with an agonist anti-GITR mAb DTA-1 (0.5 mg, i.p.) resulted in ∼50% reduction in tumour foci in the lungs, as compared with control IgG-treated mice (Fig. 8b). At time of sacrifice, real-time RT–PCR analysis of lung infiltrating cells revealed a marked reduction of *Foxp3* transcripts, and highly increased expression of *Il9* transcripts, whereas changes in other cytokine transcripts (*IL17a, IFNg, Il4*) were not remarkable (Fig. 8c). Flow cytometry analysis of lung infiltrating OT-II cells showed that Foxp3^+^ T cells, but not IL-9+ Th9 cells, were induced in tumour-bearing mice in control Ig-treated mice. However, treatment with anti-GITR mAb inhibited Foxp3^+^ Tregs, and promoted the induction of Th9 cells (0.3 to 3.5%, ∼10-fold increase in Th9 cells) (Fig. 8d). Importantly, we demonstrated that blocking IL-9 using a neutralizing anti-IL-9 mAb at the time of GITR stimulation abrogated the effects of anti-GITR mAb in protection against cancer in the lungs (Fig. 8b), thus establishing a key role for IL-9 in the anti-tumour immunity in this model.

## Discussion

In the present study we demonstrate that under iTreg-inducing conditions, GITR ligation inhibits iTregs and selectively directs the activated T cells to Th9 cells, thus subverting a potentially tolerogenic situation (Tregs) to an inflammatory one (Th9). Mechanistically, we provide evidence that GITR controls chromatin remodelling in the *Foxp3* and *Il9* loci through induction of the NF-κB family member p50 and that p50 recruits and positions histone deacetylases to the *Foxp3* locus to induce a repressive chromatin structure. Moreover, inhibition of Foxp3 allows GITR to render *Il9* locus accessible by activating the STAT6/p300 pathway and direct the transcriptional activities induced by iTreg-polarizing cytokines to the *Il9* site to support Th9 induction. Findings from our *in vivo* melanoma tumour model support Th9 cells as key effector cells in anti-tumour immunity^42^. However, whether this is due to iTreg to Th9 conversion in our model remains to be clarified in future studies. Also, inhibition of Foxp3+ Tregs by anti-GITR Ab is more prominent *in vivo* than induction of Th9 cells, the identity of those non-Th9 cells upon GITR ligation remains to be defined.

Our data suggest that iTregs and Th9 cells are developmentally related; they share key transcription factors and common induction pathways, and the locus accessibility, which is regulated by chromatin remodelling, critically determines the generation of one over the other subset. Among the T helper subsets studied thus far, Th9 cells are especially unique in that a ‘master’ transcription factor has yet been identified for Th9 induction. Instead, a multiplicity of transcription factors are known to bind to the IL-9 locus and collectively facilitate IL-9 transcription, and such transcription factors include PU.1, IRF1, IRF4, STAT5, STAT6, NFAT, GATA1, GATA3, SMAD2/3, Notch, as well as BATF, RelB/p52 (refs 9, 19). The requirement of such diverse transcription factors acting in a dynamic manner reveals the complexity of Th9 induction. Of note, SMAD2, SMAD3 and STAT5 are strongly induced by TGF-β and IL-2, which are required for the induction of Foxp3 (ref. 43). Interestingly, SMAD2, SMAD3 and STAT5 are also demonstrated for the induction of Th9 cells, as the *Il9* locus also contains binding sites for such transcription factors^29^. Moreover, under certain conditions, IL-9 can also be expressed, though at low levels, by Foxp3^+^ Tregs^22^,^24^, and the mechanism behind this is completely unknown. In the present study, we provide evidence that Foxp3 is a potent repressor of Th9 cells, and once Foxp3 is induced, it binds to the *Il9* locus, and through recruiting histone deacetylases, Foxp3 induces a ‘closed’ chromatin structure, thus rendering *Il9* locus inaccessible for transcription. This in turn reinforces the Treg programs by retaining SMAD2/3 and STAT5 at the *Foxp3* locus. However, when *Foxp3* locus is closed and the *Il9* locus becomes accessible, as in the case of GITR ligation, transcription factors that are induced by TGF-β and IL-2 then switch to the *Il9* sites to support Th9 induction. A major implication of this finding is that tolerogenic conditions that rely on iTregs can be destabilized by switching to Th9 cells. The induction of Th9 cells by GITR ligation, but not other T helper subsets, suggests that GITR plays a unique role in this regard, despite the fact that many other pathways are also shown to directly promote Th9 induction^12^,^15^,^44^.

Another important finding of our study is the discovery of histone acetylation status as a key regulatory process in induction of iTregs to Th9 cells. In contrast to histone methylation, which can be transcriptionally active or repressive depending the extent and positions of the methylated lysines, histone acetylation is generally associated with gene transcription, whereas deacetylation corresponds to gene repression^31^. Clearly, the acetylation status at the *Foxp3* and *Il9* loci under iTreg conditions is profoundly affected by p50, a key transcription factor of the canonical NF-κB pathway^45^. Traditionally, p50 forms heterodimers with RelA to activate NF-κB responsive genes^37^, as p50 itself lacks the transactivation domain and binds DNA with low affinity^46^. We showed that in activated CD4^+^ T cells, p50 binds to the *Foxp3* locus, and through recruiting histone deacetylases, p50 mediates site-specific histone deacetylation, and subsequently, closure of the *Foxp3* locus (Fig. 4). In supporting of this notion, either deficiency of p50 or addition of HDAC inhibitors prevented deacetylation of *Foxp3* locus, and rescued Foxp3 from GITR-induced repression (Figs 3b and 4e). This finding is also consistent with reports that p50 is capable of interacting with HDAC proteins in other cell types^46^,^47^. In addition, p50 can also activate STAT6, specifically STAT6 phosphorylation, and activated STAT6 are preferentially enriched at the *Il9* locus, and through recruiting p300 histone acetyltransferase, STAT6 induces histone hyperacetylation at *Il9* locus in CD4^+^ T cells, allowing Il9 locus to be accessible. On the other hand, Foxp3 can recruit histone deacetylases to *Il9* locus to mediate histone deacetylation, and consequently, closure of *Il9* locus (Fig. 5). Others reported that Foxp3 can complex with HDAC and Sirt proteins in purified natural Tregs, as revealed using proteomic approaches^48^. Our data suggest that a major function of this Foxp3/HDAC complex seems to repress *Il9* in stabilizing Foxp3^+^ Tregs. Thus, modification of histone acetylation pathways may provide additional means of altering the T-helper-cell fates.

Our data suggest that different mechanisms can be mobilized to promote Th9 induction under certain conditions. We reported before that OX40 is a powerful costimulatory molecule in promoting IL-9 producing cells when CD4^+^ T cells are activated under Th9 culturing conditions (TGF-β and IL-4)^44^, and in this setting, it is the p52/RelB pathway (non-canonical) that is critically important in Th9 induction^44^. On the other hand, GITR directs CD4^+^ T cells to Th9 cells under iTreg-inducing conditions, but requires the NF-κB family member p50 to do so (canonical pathway). As deacetylation of *Foxp3* locus is necessary for Th9 induction under iTreg conditions, p52 may act very differently from p50 in its ability to recruit histone deacetylases in epigenetically modifying the *Foxp3* locus. In fact, the finding that induction of Foxp3^+^ iTregs from p52−/− CD4^+^ T cells remains inhibited by GITR ligation appears to support this notion (Fig. 3b). Nonetheless, further studies are warranted to resolve this issue. It should be noted that the most abundant form of NF-κB in naive T cells is the p50/RelA heterodimers that are sequestered in the cytosol by an inhibitory molecule IκB^37^. Questions regarding what regulates the dynamics of p50/HDAC complex on T-cell activation, as well as its competition with P50/RelA complex in gene repression versus gene activation under various polarizing conditions also require further clarification.

Our findings may have important clinical implications. For example, in certain models where iTregs are critical to the induction of tolerance, tolerizing therapies that cater iTregs for tolerance may be subverted by GITR-mediated induction of Th9 cells. In this setting, approaches to inhibit GITR costimulation may help promote tolerance induction. Conversely, in models of anti-tumour immunity and parasitic infection, conversion of iTreg-conducive conditions to ones that favour Th9 cells can be beneficial. Thus, therapeutic approaches targeting GITR signalling pathways or GITR triggered epigenetic mechanisms may provide additional means of therapeutic intervention in immune-mediated diseases.

## Methods

### Animals

Wt C57BL/6, Rag1^−/−^, Batf^−/−^, Irf4^f/f^, Cd4-Cre and OT-II mice were purchased from The Jackson Laboratory (Bar Harbour, ME). Other mice including p52^−/−^, p50^−/−^, Sfp1^f/f^ Cd4-Cre, and *Foxp3*gfp reporter mice have been previously described^44^. Some strains were crossed with the *foxp3*gfp mice to genetically mark Foxp3^+^ Treg cells for cell sorting. Conditional deletion of *Irf4* in T cells was carried out by crossing Irf4^f/f^ with Cd4-Cre mice. Male mice at age of 8 weeks were used for all the experiment. All animals were maintained in specific pathogen free facility at Houston Methodist Research Institute in Houston, Texas. Animal use and care were approved by the Houston Methodist Animal Care Committee, in accordance with institutional animal care and use committee guidelines.

### Polarization of naive CD4^+^ T cell *in vitro*

Naive CD4^+^ T cells (CD62L^+^CD44^−^Foxp3gfp^−^) were FACS sorted with the high-speed cell sorter FACSaria, and the sorted T cells were activated with anti-CD3 (2 μg ml^−1^; clone 2C11; eBioscience) plus equal numbers of syngeneic APCs (1 × 10^5^ cells per well) in 96-well tissue-culture plates (Sigma-Aldrich). In some experiments, OT-II CD4^+^ T cells were stimulated with OVA_323-339_ (1  μg ml^−1^) presented by autologous APCs. APCs were prepared from total spleen cells by depletion of T cells with phycoerythrin–anti-CD3 (clone 2C11) and anti-phycoerythrin microbeads (Miltenyi Biotec), followed by brief treatment with mitomycin C (50  μg ml^−1^; Sigma-Aldrich) before each experiment.

To induce Foxp3^+^ iTregs *in vitro*, sorted naïve CD4^+^ T cells were activated in the presence of TGF-β1 (3 ng ml^−1^) and IL-2 (10  ng ml^−1^)^44^. All recombinant cytokines were from R&D Systems (Minneapolis, MN). To deliver GITR costimulation to activated T cells, an agonist anti-GITR antibody DTA-1 (10 μg ml^−1^) or a His-tagged recombinant mouse GITR Ligand (200 ng ml^−1^) (2177-GL-025, R&D Systems) were added into the cultures, and in the case of His-tagged GITRL, anti-His mAb (10 μg/ml, MAB050, R&D Systems) were used to cross-link surface bound GITRL. In some experiments, naïve CD4^+^ T cells were activated as described above, alone with various concentrations of HDAC1 inhibitor CI-994 (CI), Sirt1 inhibitor EX-527 (EX) (selleckchem), p300/CBP inhibitor 1 (p300i-1), pan-HDACs inhibitor Trichostatin A (TSA), sodium butyrate (NaB) (Calbiochem), and p300 HAT activator (CTPB) (Sigma), as indicated in the text. Naive CD4^+^ T cells were polarized under such conditions for 1–3 days, and at these time points, activated T cells were collected for analyses by intracellular staining, ChIP and Immunoblot methods.

### Intracellular staining

For cytokine staining, CD4^+^ T cells activated under iTreg-polarizing conditions were briefly restimulated for 4 h with phorbol 12-myristate 13-acetate (50 ng ml^−1^) and ionomycin (550 ng ml^−1^; Sigma-Aldrich) in the presence of GolgiStop (BD PharMingen). For retrovirus-transduced T cells, cells were fixed and permeablized with Cytofix/Cytoperm solution (BD PharMingen) or with foxp3 staining buffer set (eBioscience), and then stained with fluorochrome-conjugated anti-IL-9 (RM9A4, 1:1,000; BioLegend), anti-Foxp3 (FJK-16s, 1:200; eBioscience), anti-IL-4 (11B11, 1:100; BD Pharmingen), anti-interferon-γ (XMG1.2, 1:200; BioLegend), anti-IL-17A (TC11, 1:200; BioLegend) and anti-IL-22 (Poly5164, 1:20; BioLegend) antibodies according to the manufacturers’ instructions. All samples were acquired with LSRII (Beckton Dickinson) and data were analysed with FlowJo v10 software (Tree Star)^44^.

### Quantitative RT–PCR

Cellular RNA was extracted with the RNeasy mini kit (Qiagen) and was reverse-transcribed into cDNA by the ABI PRISM TaqMan reverse transcription method. Expression of genes of interest and of *Gapdh* was assessed by Simplex RT–PCR with probes labelled with 5-carboxyfluorescein and VIC fluorescent dye (Applied BioSystems). All the probe sets were from Applied BioSystems (Supplementary Table 1). Transcription of target genes was calculated according to the 2^−ΔΔCT^ method as described by the manufacturer (ABI PRISM 7700 user bulletin; Applied BioSystems) and was presented in arbitrary units as previously reported^49^.

### Immunoblot analysis

Protein extracts were resolved by SDS–PAGE, transferred onto an Immunobilon membrane, and analysed by immunoblot with the following specific antibodies: anti-p105/p50 (ab32360, 1:5,000), anti-NF-κB p65 (RelA) (ab16502, 1:5,000), anti-p300 (ab3164, 1:1,000), anti-HDAC2 (ab7029, 1:1,000), anti-Sirt1 (ab110304, 1:1,000; all from Abcam); anti-p100/p52 (4882, 1:1,000), anti-RelB (4922, 1:1,000), anti-Histone H3 (4499, 1:2,000), anti-β-actin (12262, 1:2,000), anti-Batf (8638, 1:1,000), anti-PU.1 (2258, 1:1,000), anti-Phospho-STAT5 (9359, 1:1,000), anti-Smad2 (5339, 1:1,000), anti-Phospho-Smad2 (3108, 1:1,000), anti-Smad3 (9523, 1:1,000), anti-Phospho-Smad3 (9520, 1:1,000), anti-Sirt7 (5360, 1:1,000), anti-HDAC1 (2062, 1:1,000; all from Cell Signaling Technology); and anti-STAT5 (SC-835X, 1:3,000), anti-STAT6 (SC-981X, 1:4,000), anti-phospho-STAT6 (SC-11762X, 1:3,000), anti-IRF4 (sc-6059, 1:1,000; all from Santa Cruz Technology). Horseradish peroxidase (HRP)-linked antibody to mouse IgG (7076, 1:2,000), HRP–linked antibody to rabbit IgG (7074, 1:2,000; both from Cell Signaling Technology) and HRP-linked antibody to goat IgG (sc-2768, 1:2,000; Santa Cruz Technology) were used as secondary antibodies. The expression of target molecules was detected by chemoluminescence method. Images has been cropped for presentation; full-size immunoblots are provided in Supplementary Fig. 7.

### Retrovirus-mediated gene transfer

The cDNA fragments encoding mouse PU.1, IRF4, batf, and Foxp3 (with or without a FLAG tag) were amplified by PCR and further cloned into the pMYs–IRES–EGFP retroviral vector (Cell Biolabs). Retroviral particles were prepared by transfection of packaging Plat-E cells according to the manufacturer’s recommendations (Cell Biolabs). For transduction of T cells, naive CD4^+^ T cells were first activated for 24 h with mouse CD3/CD28 T-cell Expander Dynabeads for 24 h (Invitrogen), followed by incubation with freshly prepared retroviral particles at 32 °C in the presence of 10 μg ml^−1^ polybrene (Sigma-Aldrich) by centrifugation for 2 h at 780 *g* and. After centrifugation, cells were further cultured for 6 h at 32 °C, followed by culture of the transduced T cells under iTreg-polarizing conditions for additional 3days in complete 1640 medium at 37 °C. The polarization of CD4^+^ T cells into Th9 cells was assessed by intracellular cytokine staining.

### Co-immunoprecipitation assay

Naïve CD4^+^Foxp3^−^ T cells (WT or S*tat6*^*−/−*^) were polarized under iTreg-polaring conditions (TGF-β plus IL-2) plus GITR costimulation, and 24 or 48 h later cells (1 × 10^7^ cells) were collected in IP lysis buffer (87788; Thermo Scientific). Subsequently, cell lysate was incubated with 5 μg anti-STAT6 (SC-981X), anti-p50 (sc-1192; both from Santa Cruz Technology) or purified goat IgG (02–6202; Life Technologies) at 4 °C overnight and then with Protein G agarose beads (16–201, 40 μl; EMD Millipore) for additional 4 h. The agarose beads were collected by centrifugation and then washed four times with IP lysis buffer. Proteins were eluted in reducing sample buffer (39000; Thermo Scientific) and analysed by immunoblot. FLAG-Foxp3 retrovirus was transduced into naive CD4^+^Foxp3^−^ T cells (1 × 10^7^ cells) polarized under iTreg-culturing condition with GITR costimulation, and 48 h later, cells were collected and processed as above except that anti-FLAG antibody (F1804; Sigma-Aldrich) and normal mouse IgG (sc-2025; Santa Cruz Technology) were used for immunoprecipitation.

### Chromatin immunoprecipitation assay

Chromatin was extracted from naive CD4^+^Foxp3^−^ T cells (1 × 10^6^ cells) polarized for 24–72 h under various conditions after fixation with formaldehyde. Anti-Histone H3ac (39139, 7 μl), anti-Histone H4ac (39243, 6 μl), anti-Histone H3 (trimethyl K9) (61013, 7 μl), anti-Histone H3 (dimethyl K27) antibody (39245, 5 μl; all from Active Motif), anti-HDAC1 (ab7028, 5 μg), anti-Smad3 (ab28379, 5 μg), anti-Histone H3 (trimethyl K27) (ab6002, 5 μl), anti-Histone H3 (acetyl K27) (ab4729, 3 μl), anti-Histone H3 (acetyl K9) (ab4441, 4 μl; all from Abcam), anti-dimethyl-Histone H3 (diMe-Lys9) (D5567, 5 μl; Sigma- Aldrich), anti-p50 (sc-1192X, 5 μg), anti-p300 (sc-585X, 5 μg), anti-STAT6 (SC-981X, 5 μg), control mouse IgG (sc-2025, 5 μg; all from Santa Cruz Technology) and purified goat IgG (02–6202, 5 μg; Life Technologies) were used for the immunoprecipitation of chromatin with an EZ ChIP kit (17–371; EMD Millipore) according to the manufacturer’s instructions. The precipitated DNA was measured by real-time PCR using SsoAdvanced Universal SYBR Green Supermix (172–5275; Bio-Red). Real-time PCR primer sequences are listed in Supplementary Table 2. Data are presented as relative binding based on normalization to input DNA^50^.

### Melanoma tumour model

CD4^+^ Foxp3-GFP^−^ T cells were sorted by flow cytometry from the spleens cells of OT-II foxp3gfp mice and p50^−/−^ OT-II foxp3gfp mice. For the induction of tumour in Rag1^−/−^ mice, 4 × 10^6^ of CD4^+^ Foxp3GFP^−^ cells together with 3 × 10^5^ B16-OVA cells were injected intravenously into Rag1^−/−^ mice. Then the mice were received three doses (0.5 mg, i.p.) of DTA-1 (*n*=14) or control IgG (*n*=14) every 5 days starting at day 5 after B16 tumour cells inoculation. *In vivo* neutralization of IL-9 (*n*=7) was carried out by i.p. injection of 200 μg anti-IL-9 (MM9C1; BioXcell) on days 0 and every 3 days after tumour cell inoculation. Lung melanoma tumour nodules were determined 18 days later blindly^18^. Lymphocytes isolated from spleen and lungs were briefly stimulated with PMA and ionomycin in the presence of GolgiStop for 4 h for intracellular Foxp3 and IL-9 staining. Lung CD4 T cells were also purified for assessment by quantitative RT–PCR.

### Statistics

Data were prepared as mean±s.d. and analysed with Prism version 6.04 for Windows (GraphPad Software). Measurements were performed using unpaired two-tailed Student’s *t*-test. A *P* value<0.05 was considered significant.

## Additional information

**How to cite this article:** Xiao, X. *et al*. GITR subverts Foxp3^+^ Tregs to boost Th9 immunity through regulation of histone acetylation. *Nat. Commun.* 6:8266 doi: 10.1038/ncomms9266 (2015).

## Supplementary Material

Supplementary Information Supplementary Figures 1-7 and Supplementary Tables 1-2
