# Supplementary material for: GITR subverts Foxp3+ Tregs to boost Th9 immunity through regulation of histone acetylation
Source: Nat Commun. 2015 Sep 14;6:8266. doi: 10.1038/ncomms9266 (PMC4570275; doi:10.1038/ncomms9266)
Supplement: Supplementary Information — Supplementary Figures 1-7 and Supplementary Tables 1-2 [file ncomms9266-s1.pdf]

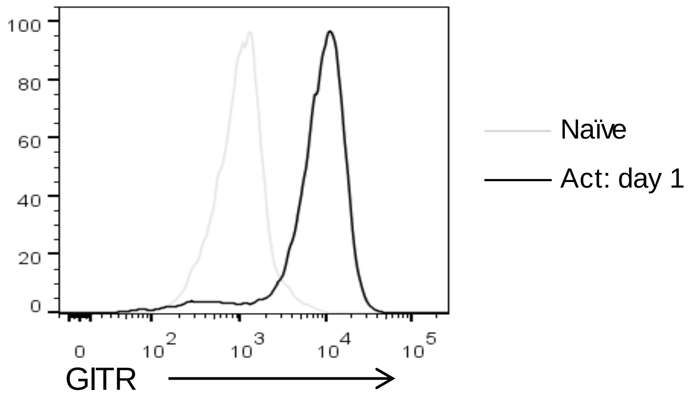

Supplementary Fig 1. GITR expression by naïve and activated CD4+ T cells

Naïve CD4+ T cells were activated with anti-CD3 plus APCs for 24 hrs (Act: day 1); expression of GITR on the cell surface was analyzed by flow cytometry and compared with naïve unstimulated CD4+ T cells (Naïve).

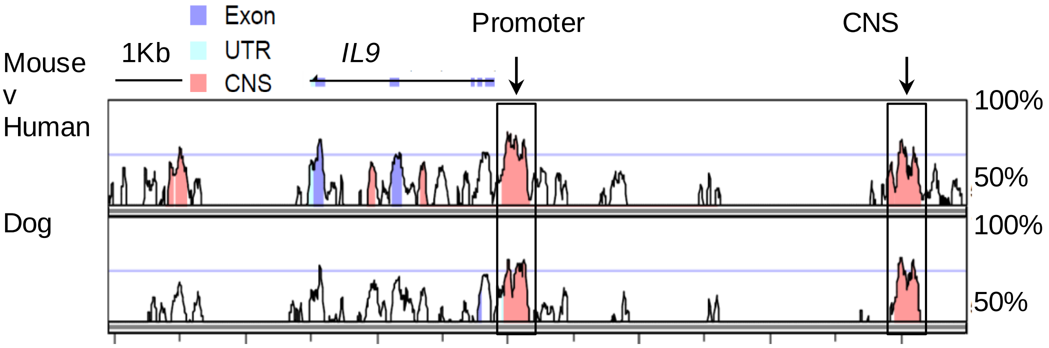

Supplementary Fig 2. Schematic plot of the *Il9* gene locus indicating the intron–exon, promoter, and conserved non-coding regions.
VISTA schematic plot showing sequence similarity of conserved non-coding sequence (CNS) sites (pink) between mouse Il9 vs human and dog Il9 DNA, presented relative to their positions in the mouse genome (horizontal axis). The promoter and CNS regions are located at –1 b and –6 kb, respectively, from the transcriptional starting site of the mouse *Il9*.

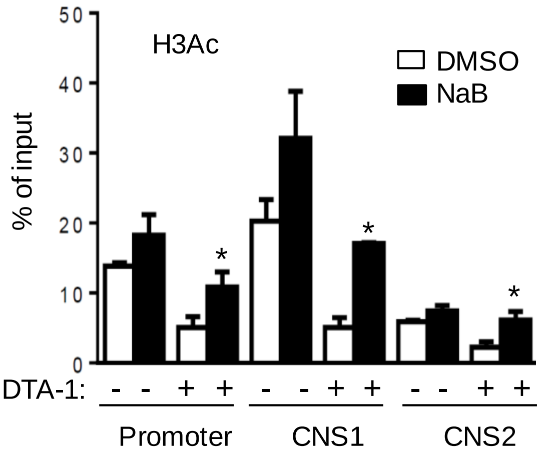

Supplementary Fig 3. The HDAC inhibitor sodium butyrate prevents GITR-mediated hypo-acetylation of *Foxp3* locus

ChIP analysis of H3 acetylation status of Foxp3 locus, including the promoter, CNS1, and CNS2 regions in naive CD4+ T cells activated under iTreg-polarizing conditions and GITR ligation in the presence or absence of HDAC inhibitor sodium butyrate (NaB, 250μg/ml) for 2 days. Data are representative of two independent experiments. * $p < 0.05$.

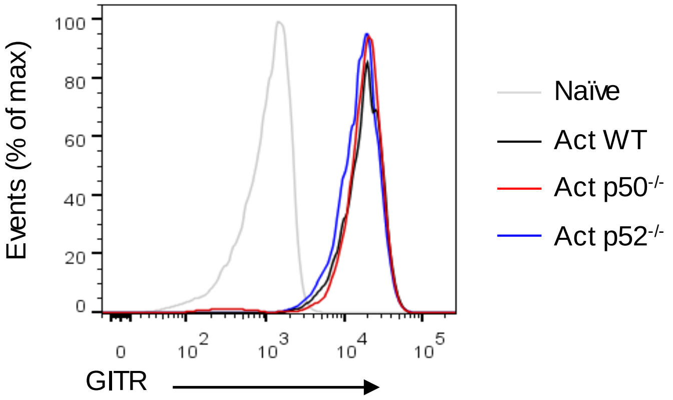

Supplementary Fig 4. Wt B6, p50$^{-/-}$, and p52$^{-/-}$ CD4+ T cells express similar levels of GITR on the cell surface upon activation

GITR expression by Wt, p50$^{-/-}$and p52$^{-/-}$ CD4+ T cells, left unstimulated (Naive; resting cells) or activated for 2 days under iTreg-polarizing conditions (Act) was analyzed by flow cytometry and shown. Similar patterns were observed on day 3 and day 4 following activation

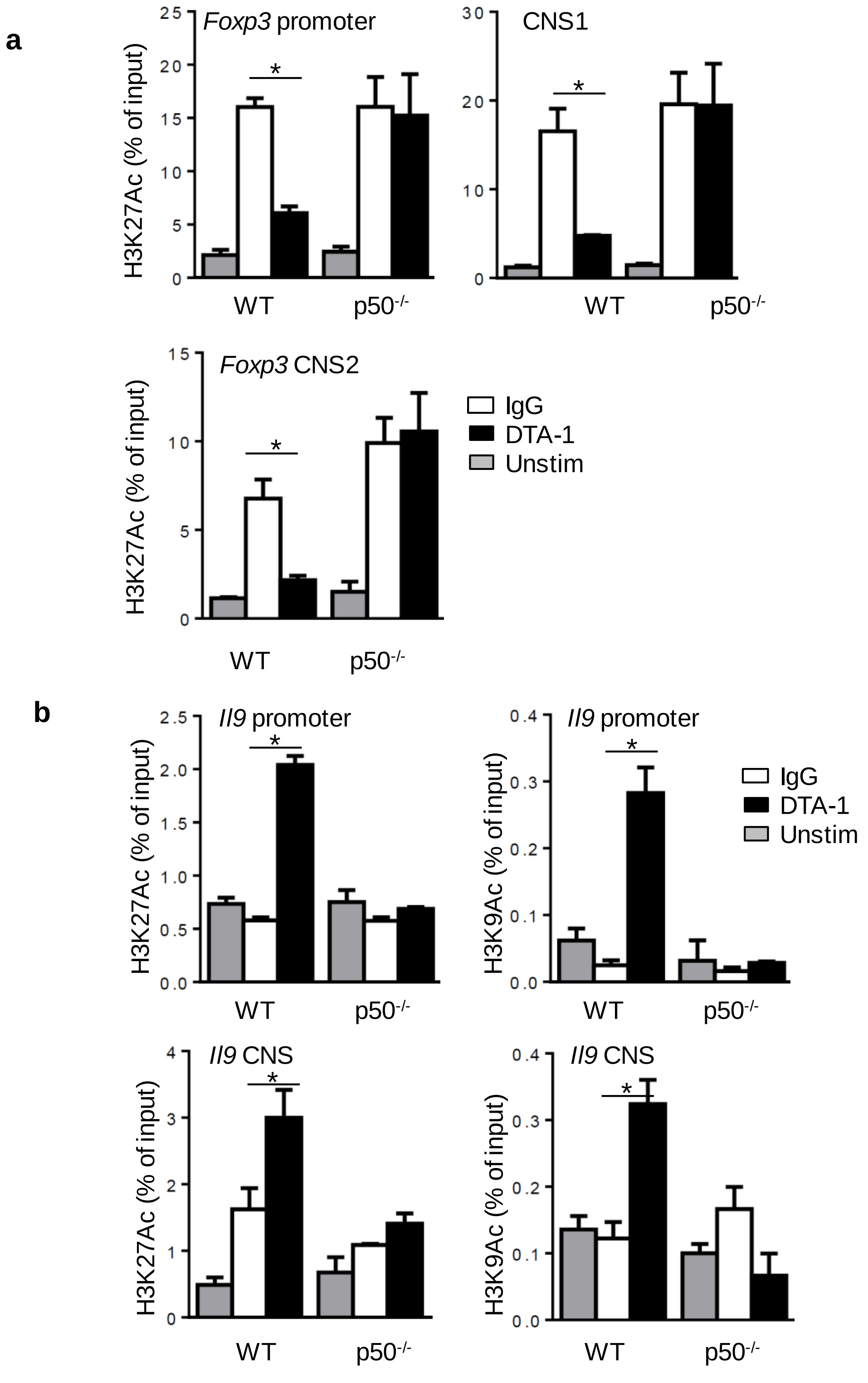

Supplementary Fig 5. GITR-induced differential chromatin remodeling responses at the *Foxp3* and *Il9* loci require p50.

ChIP analysis of H3K27AC and H3K9Ac modifications at *Foxp3* (**a**) and *Il9* (**b**) promoter and CNS regions from Wt B6 and p50$^{-/-}$ CD4+ T cells activated under iTreg-polarizing conditions for 2 days in the presence of DTA-1 or Ctrl IgG. Data are representative of two independent experiments.

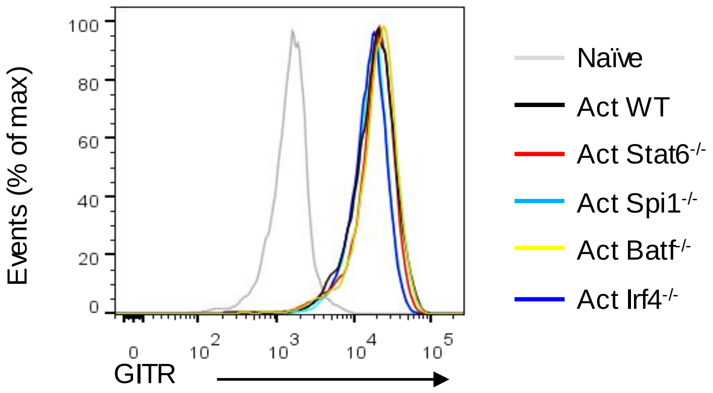

Supplementary Fig 6. Wt B6, Spi1$^{-/-}$, Batf $^{-/-}$, IRF4$^{-/-}$ and Stat6$^{-/-}$ CD4+ T cells express similar levels of GITR on the cell surface upon activation

GITR expression by CD4+ T cells from Wt B6, Spi1$^{-/-}$, Batf $^{-/-}$, IRF4$^{-/-}$ and Stat6$^{-/-}$ mice, either left unstimulated (Naive; resting cells) or activated for 2 days under iTreg-polarizing conditions (Act), was analyzed by flow cytometry and shown. Similar patterns were observed on day 3 and day 4 following activation.

Fig. 2A

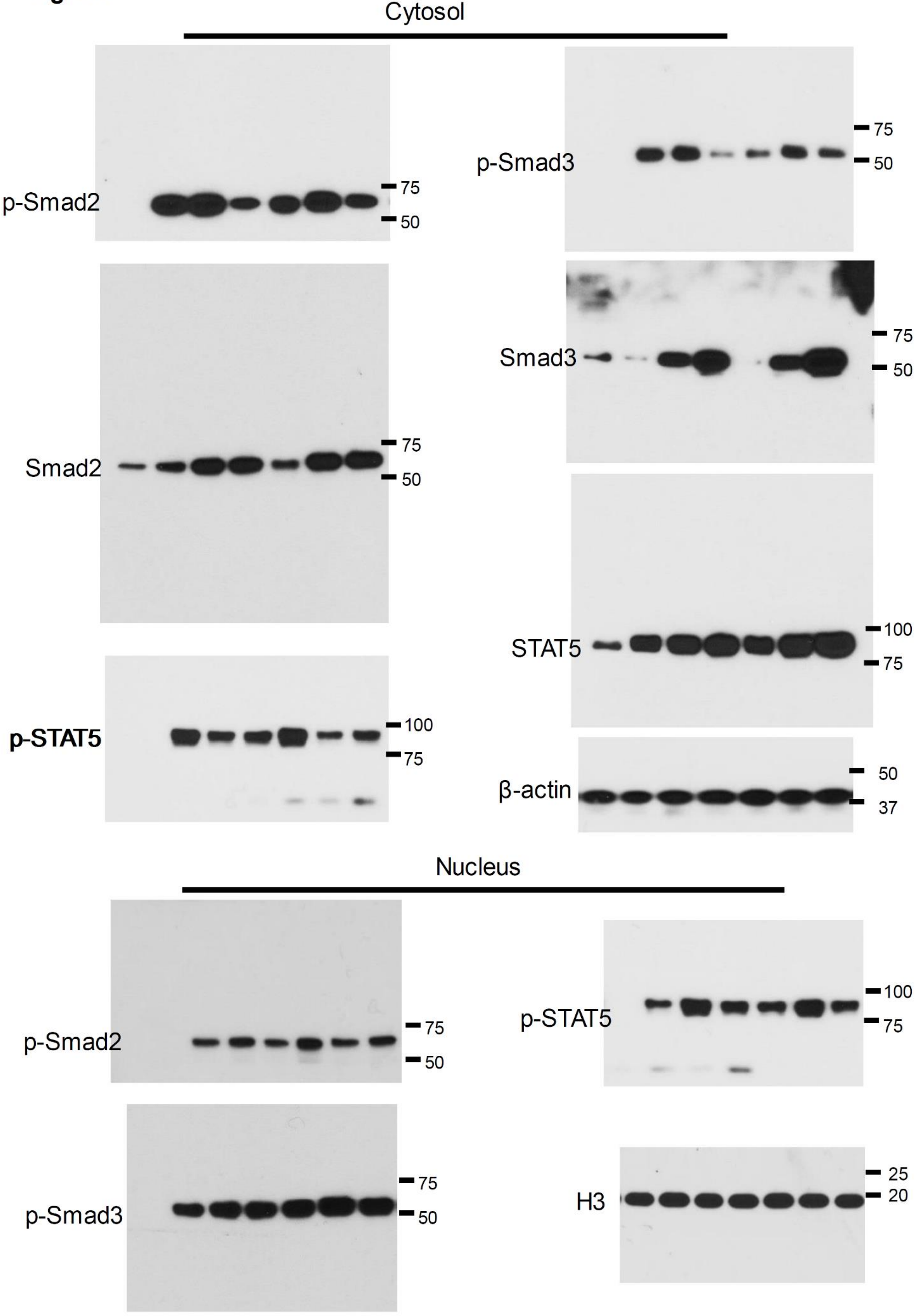

Supplementary Fig 7. Immunoblots (1)

Fig. 3A

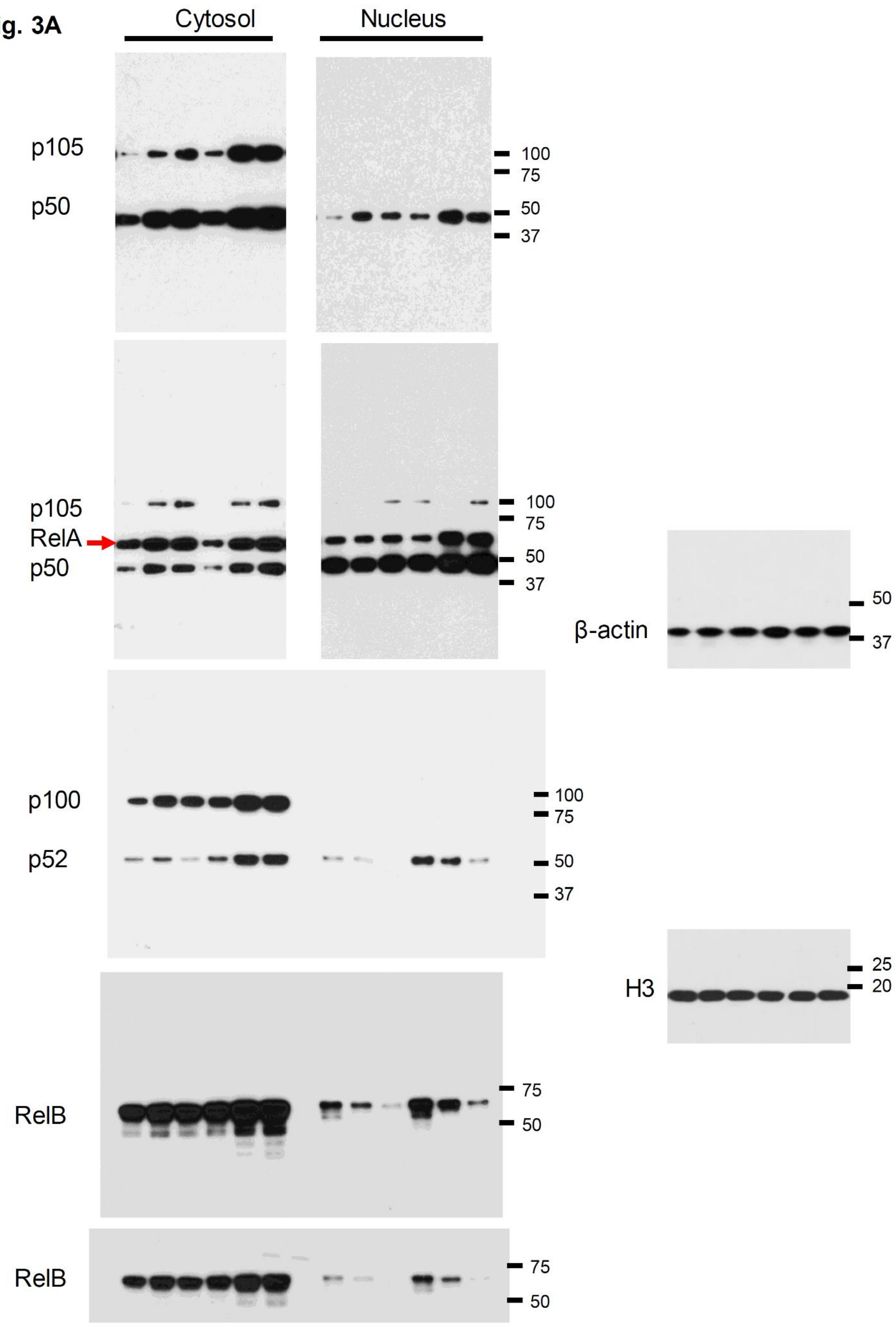

Supplementary Fig 7. Immunoblots (2)

Fig. 4C

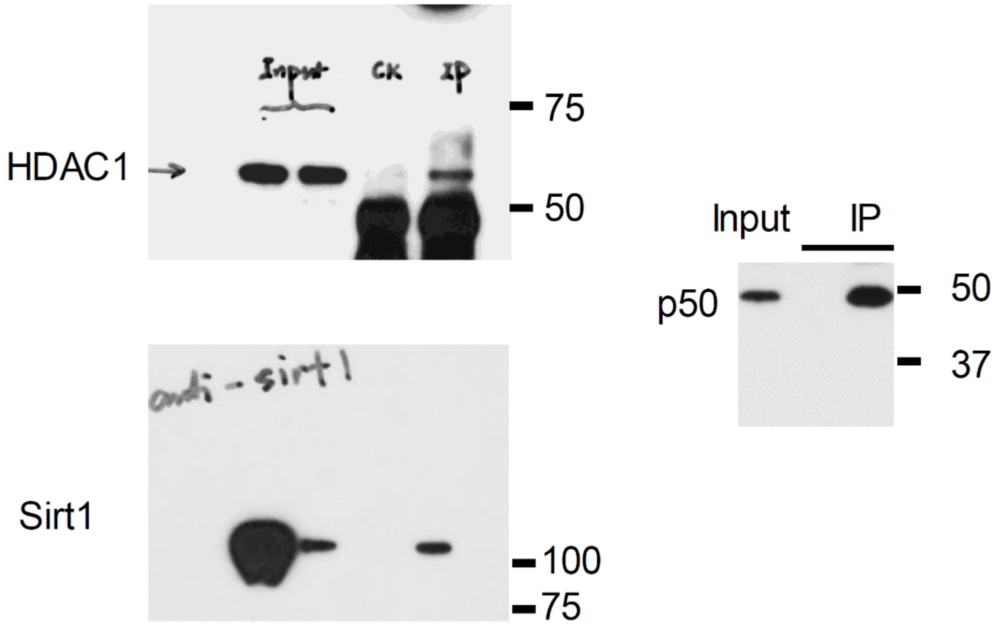

Fig. 5E

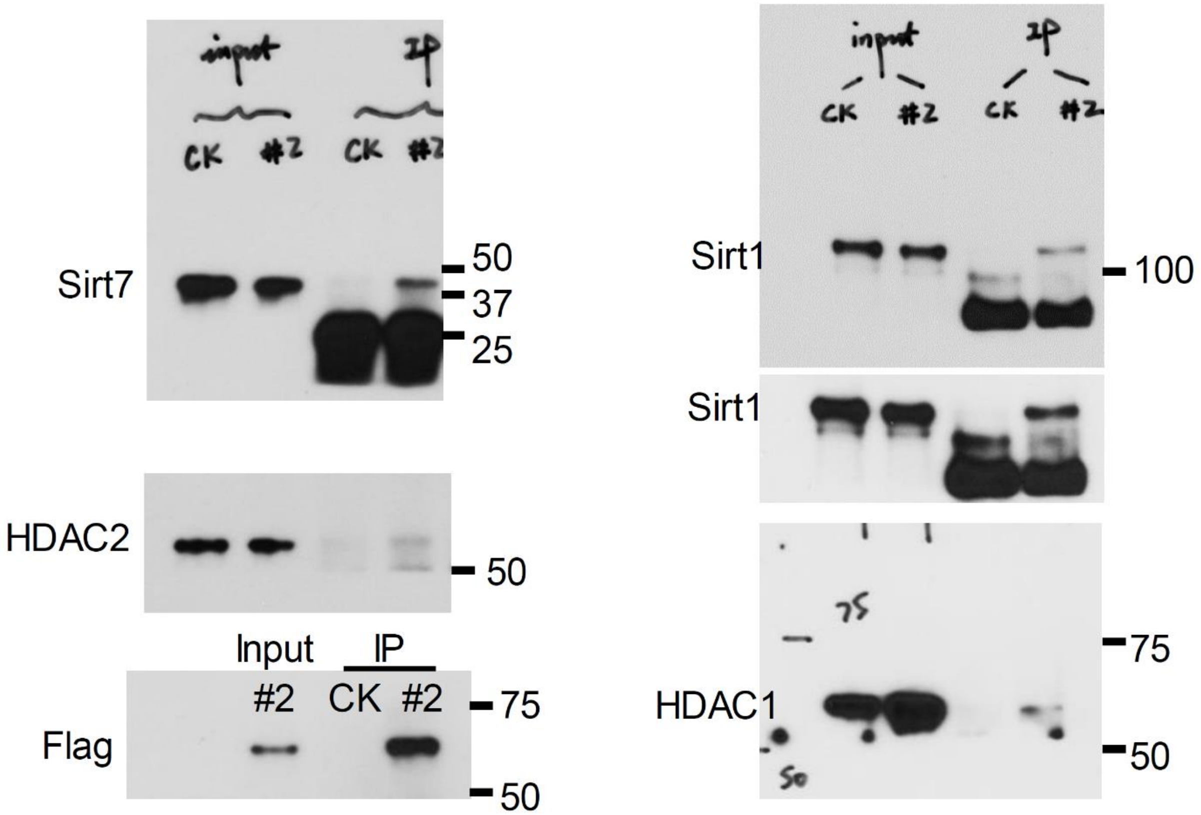

Supplementary Fig 7. Immunoblots (3)

Fig. 6A

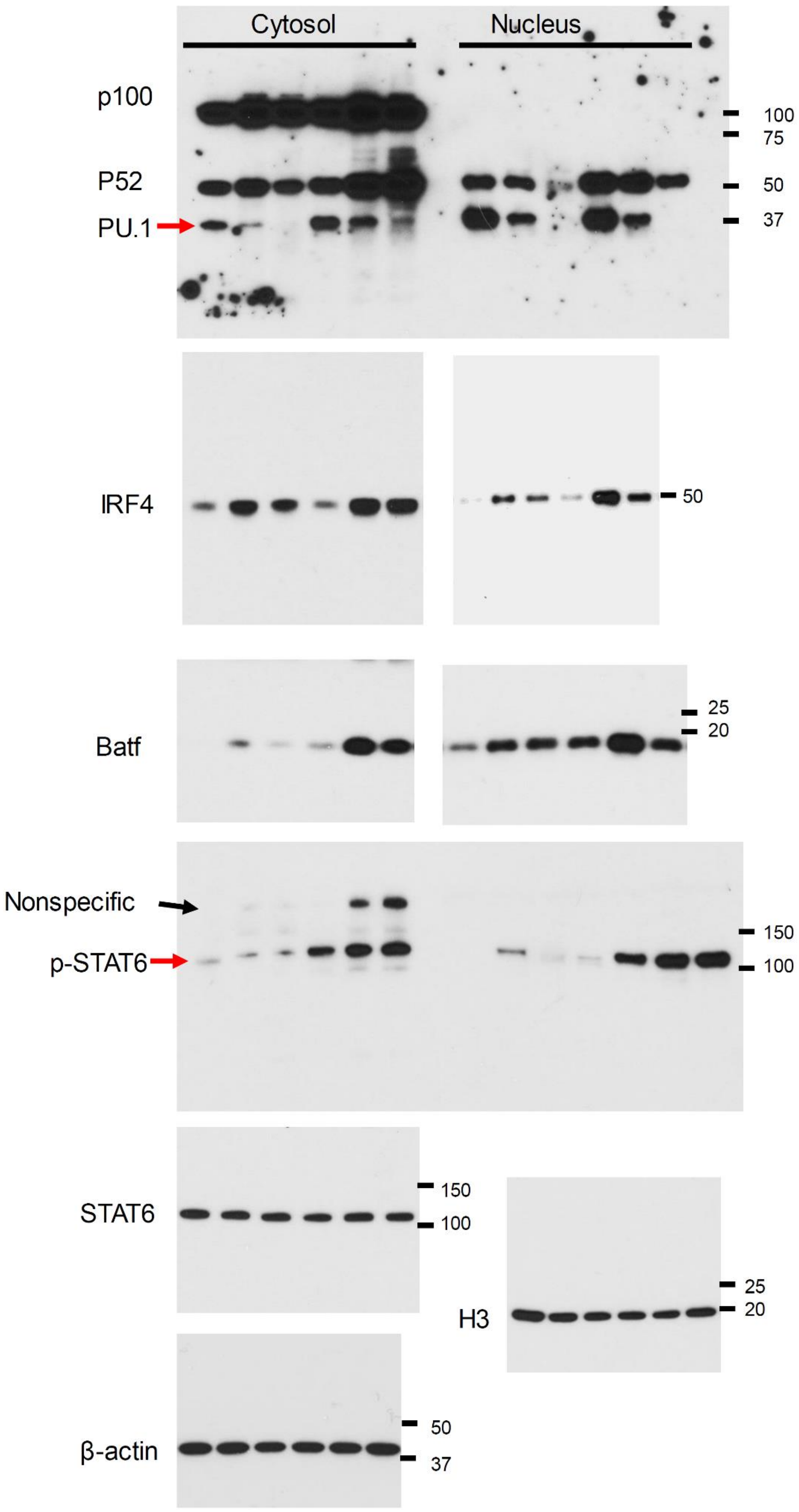

Supplementary Fig 7. Immunoblots (4)

Fig. 6C

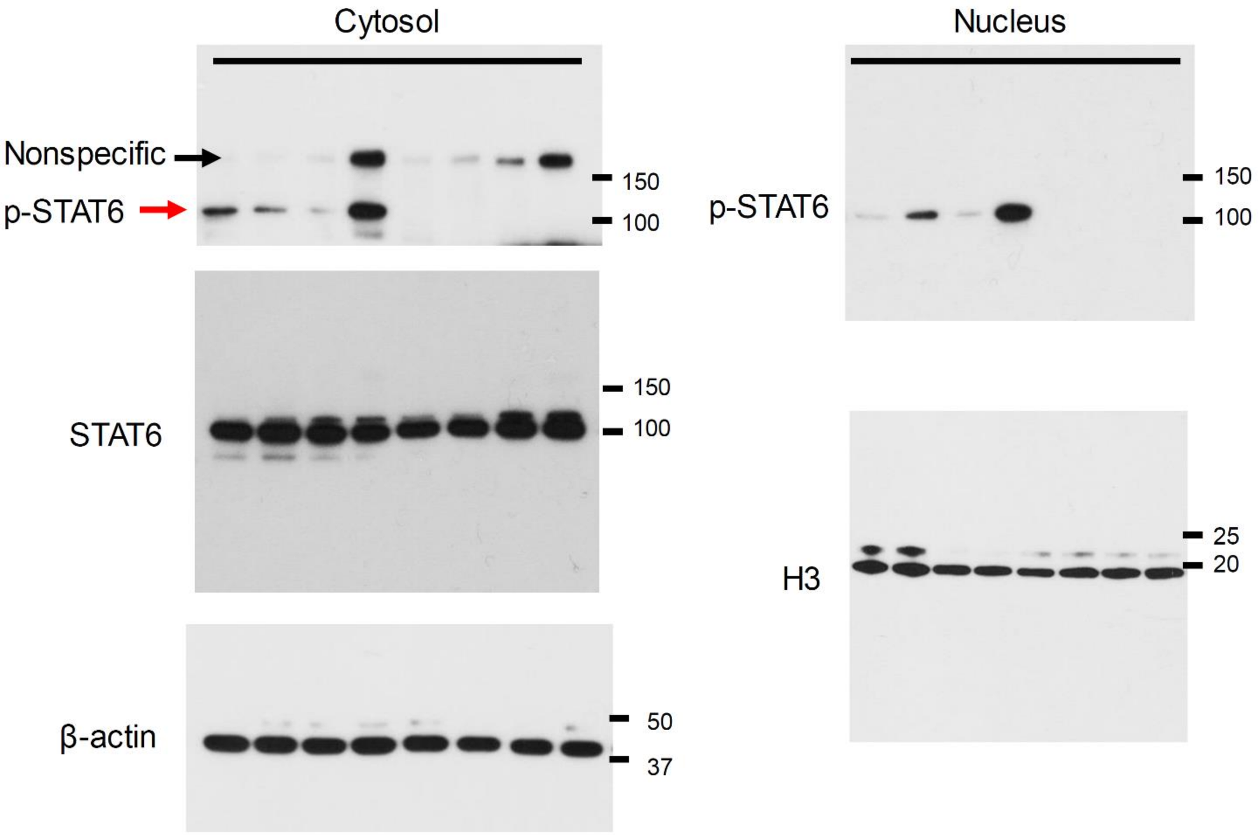

Fig. 7D

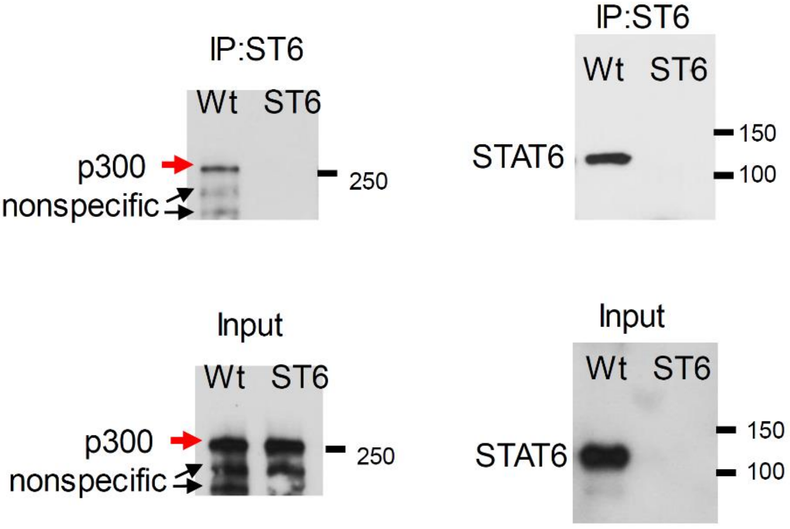

Supplementary Fig 7. Immunoblots (5)

Supplementary Table 1. Real-time PCR Primers for figure 8

Gene Name	Catalogue # (Applied Biosystems)
Gapdh	4352339E
Foxp3	Mm00475162_m1
Il9	Mm00434305_m1
Il17a	Mm00439618_m1
Ifng	Mm01168134_m1
Il4	Mm00445259_m1

Supplementary Table 2. Real-time PCR Primers for ChIP assays.

Primer Name	Primer Sequence
Foxp3_promoter_forward	CTGAGGTTTGGAGCAGAAGGA
Foxp3_promoter_reverse	TCTGAAGCCTGCCATGTGAA
Foxp3_CNS1_forward	ACTTAGTTTATGAGCATGCATGTTCTTC
Foxp3_CNS1_reverse	TGAGATCCCACACCATCTTCTG
Foxp3_CNS2_forward	GTTGCCGATGAAGCCCAAT
Foxp3_CNS2_reverse	ATCTGGGCCCTGTTGTCACA
Foxp3_promoter_kB site_forward	CTGGCGAAAGTGGCAGAG
Foxp3_promoter_kB site_reverse	CTGGCATTTAGGATGAATGGTATTAG
Foxp3_CNS1_kB site_forward	ATTTACCCCAGCCACGAGAC
Foxp3_CNS1_kB site_reverse	ACTCTGACACAACATCTCTGTGAGG
Foxp3_CNS2_kB site_forward	CCCCGATAGGAAAACATATTCTATGT
Foxp3_CNS2_kB site_reverse	AGCCAGCTTCCTGCACTGT
Foxp3_CNS1_Smad3 site_forward	CCC ATGTTG CTTCCAGTCTCCTTTAT GG
Foxp3_CNS1_Smad3 site_reverse	AGGTACAGAGAGGTTAAGAGCCTGGGT
Il9_promoter_forward	TTCTCACTAATGTGGAGGGGA
Il9_promoter_reverse	GTCGGGTTCTGAAATACTAAAGG
Il9_CNS_forward	GTGCTGAAGGATTCTTGGAA
Il9_CNS_reverse	ACAAAATGACCCCCAAAATT
Il9_promoter_foxp3 site_forward	TTCTCACTAATGTGGAGGGGA
Il9_promoter_ foxp3 site_reverse	GTCGGGTTCTGAAATACTAAAGG
Il9_CNS_ foxp3 site_forward	GGGGTCATTTTGTGTTGTGAC
Il9_CNS_ foxp3 site_reverse	TTGTTCCAAAGATAAAGCTCCAC
Il9_Smad3 site_forward	CTCAATTGGCCTCAACTTACAG
Il9_Smad3 site_reverse	GAACTCAGTCTACCAGCATCTTC
Il9_Promoter_STAT6 site_forward	GAACCCGACTATTTGAAGAGC
Il9_Promoter_STAT6 site_reverse	TTTTTTCTCTGTAAGTTGAGGCC
Il9_CNS_STAT6 site_forward	CACTAGGCTTTTAATCACAAACTAAGTG
Il9_CNS_STAT6 site_reverse	TGCAGATACTATCTTTTGCAATAAATG
